# Predicting Micropollutant Removal in Wastewater Treatment Based on Molecular Structure: Benchmark Data and Models

**DOI:** 10.1021/acs.est.5c09314

**Published:** 2025-10-08

**Authors:** José Andrés Cordero Solano, Jasmin Hafner, Michael S. McLachlan, Heinz Singer, Kathrin Fenner

**Affiliations:** 1 Department of Environmental Chemistry, 28499 Swiss Federal Institute of Aquatic Science and Technology (Eawag), Dübendorf 8600, Switzerland; 2 Department of Chemistry, University of Zürich, Zürich 8057, Switzerland; 3 Department of Environmental Science, Stockholm University, Stockholm 106 91, Sweden

**Keywords:** micropollutants, wastewater treatment, QSAR, model, data set, breakthrough

## Abstract

Models to predict the environmental fate of micropollutants are needed for alternatives assessment and safe-by-design efforts. Wastewater treatment plants (WWTPs) are the main barrier to prevent micropollutants from entering receiving water bodies, and WWTP breakthrough is an important indicator of chemical persistence. State-of-the-art models to predict breakthrough are limited by their need for first-order degradation rate constants, a metric that is often unavailable. Here, we build models that predict removal in conventional treatment directly from the chemical structure using data from field-scale monitoring for over 1000 chemicals. The best predictions were achieved using substructure-based fingerprints (i.e., MACCS) and random forests, and identified influential substructures agree with structural moieties relevant for biotransformation. We show that our models are more reliable than existing process-based models used in EU and US regulatory contexts, making them important contributions to the *in silico* toolbox for alternatives assessment, the design of more benign chemicals in industrial research and development, and even exposure modeling in a risk assessment context. Moreover, our data sets along with our extensive systematic evaluation of different curation criteria and the scripts to reproduce it are key for future model advancement. Our model is publicly available (pepper-app) along with the training data and the scripts to reproduce the data curation process (github.com/FennerLabs/pepper).

## Graphical abstract

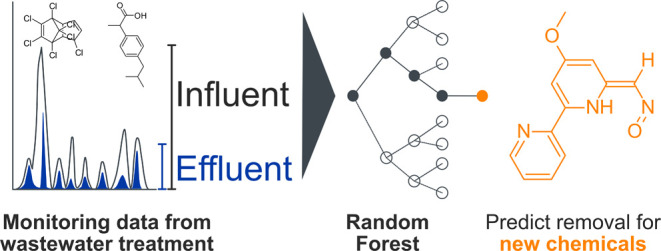

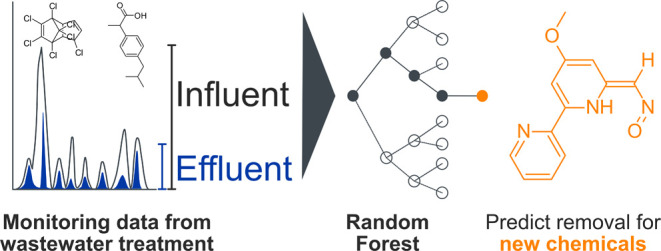

## 1 Introduction

Continuous release of harmful, wastewater-borne substances from wastewater treatment plants (WWTPs) remains a global issue. There is an urgent need for strategies to mitigate the impact of WWTP effluent on human health and the environment. Besides advanced treatment, useful strategies include alternatives assessment and safe-by-design initiatives.^1–3^ Both strategies would highly benefit from models to predict removals of individual chemicals in WWTPs, but sufficiently accurate models to do so are missing.

Examples of state-of-the-art tools for predicting chemical removal in WWTPs are the STP model in EPI Suite (i.e., STPWIN^4^) and SimpleTreat,^5^ both widely used in risk assessment and regulatory settings.^6^ These models have a strong mechanistic foundation and effectively describe the various processes that influence the fate of chemicals within treatment plants.^7–10^ However, their performance relies on the accuracy with which the properties that govern these processes can be described. In particular, accurate predictions for polar substances depend on a sound knowledge of biotransformation rate constants, but high-quality experimental biotransformation data are lacking for most chemicals in commerce, and would, in any case, not be available in a safe-by-design context.^11,12^

The most popular QSARs for predicting biotransformation are the EPI Suite BIOWIN models, which are also used to estimate biotransformation rates in STPWIN. BIOWIN models are trained on a small data set of biodegradation ratings derived from expert judgment.^13^ While recent studies continue using these data sets to develop predictive models,^14,15^ we and others believe that models should use experimental data rather than expert judgment. Notably, Wang et al.^16^ developed linear regression-based QSAR models based on rate constants derived from batch experiments with activated sludge from a WWTP. Their models provided new insights into biotransformation mechanisms under both aerobic and anaerobic conditions, but they were validated with only 10 chemicals. Differently, Nolte et al.^17^ used monitoring data from full-scale WWTPs for 69 chemicals to derive biotransformation rate constants and to build models for predicting removal. Both studies are limited by the small number of substances covered. Later, Chirico et al.,^18^ profiting from advances in high-resolution mass spectrometry (HRMS), were able to combine data for over 300 compounds to develop linear models with limited accuracy. The authors acknowledged the challenge of creating a general model due to the heterogeneity of the data and the possibility that these relationships are nonlinear.

Given the complex relationship between chemical structure, microbial biotransformation, and other processes occurring in wastewater treatment, machine learning (ML) has emerged as a promising alternative to the traditional multiple linear regression used in QSAR modeling as it can capture nonlinear relationships and prevent overfitting.^19–21^ For example, Zhang et al.^22^ demonstrated high performance (i.e., 85.1% accuracy) using ML to classify compounds as readily or not readily biodegradable. However, this model is not suited for more refined persistence and exposure assessment because it does not provide a continuous metric such as percent removal or a half-life.

Here, we address the limitations of previous studies (see Section S1 for additional context) by using ML and monitoring data from WWTPs to develop predictive models for removal of chemicals in WWTPs. Our goal is to establish a robust benchmark for future modeling advancements. To this end, we collate an unprecedentedly large set of removal data from several large-scale WWTP monitoring campaigns and explore a variety of algorithms and molecular representations to illustrate the applicability and current limits of ML for predicting removal in WWTPs. We do this in a fully transparent way by developing an open-source library and providing our carefully curated database for others to use and explore alternative modeling approaches.

## 2 Methods

### 2.1 Description of Available Data

The data used in this study consist of information on 1153 unique chemical substances monitored in 44 WWTPs across Australia, Sweden, and Switzerland; all these plants employ conventional treatment with activated sludge. These data were compiled from four independent data sets (i.e., AMAR, AUS, SNF and SWE2), which cover overlapping sets of chemical substances: out of the 1153 substances, 751 are unique to one of the data sets and 402 are found in two or more data sets. Further details about the sources and experimental procedures used to generate each data set are provided in the Supporting Information (SI) Section S2. The data sets also vary in their chemical identification and quantification methods: specifically, SNF uses reference standards for quantification and identification (level 1 confidence as described Schymanski et al.^23^), whereas for the other data sets structural annotation is done by library spectrum match and quantification is based on area (level 2 confidence^23^). A table summarizing key information on these data sets is provided in the SI Table S2, and the complete database is available in the ERIC open repository (EAWAG Research Data Institutional Collection: ERIC:QSAR-ready-micropollutants-dataset) and fully documented code in GitHub (github.com/FennerLabs/pepper).

Our target variable for modeling is breakthrough, which is defined as the quotient of the concentration (C) detected in the effluent and the concentration detected in the influent for each substance in each of the WWTPs (eq 1); thus, breakthrough may be interpreted as the fraction of each substance that is not removed during treatment.

$$Breakthrough\left(B\right)=\frac{C\left(\mathrm{Effluent}\right)}{C\left(\mathrm{Influent}\right)}$$ 1

The goal of the model is to predict an expected breakthrough per substance and therefore the median breakthrough across all plants was selected as the target end point.

### 2.2 Model Development

A computational workflow, PEPPER (pepper-lab·PyPI), was developed as an open-source library to build all models in this study. One of the benefits of PEPPER is that all data processing methods and models developed are documented in detail so that users can reproduce our work entirely. Further details on PEPPER are provided in the SI Section S3. Models were developed as follows: First, we used nested cross validation to build and compare among preliminary models (detailed methods are provided in the SI Section S8 and results in Section 3.2). Then, we retrained the best performing models and evaluated their performance to systematically estimate the impact of different curation criteria (detailed methods in Section 2.3 and SI Section S11). After selecting the best model with the best training set, we optimized its hyperparameters (SI Section S12). To estimate the applicability domain (AD) of this model, we tested different strategies (e.g., similarity-based and ensemble-variance-based) (detailed methods in SI Section S13), and to provide a mechanistic interpretation of the best set of descriptors we used SHAP (SHapley Additive exPlanations) (results in Section 3.4). Finally, we compared the performance of our models with the STPWIN model of the EPI Suite and applied the model to a much wider data set of over 14’000 commercial chemicals (results in Section 3.5).

### 2.3 Curation of the Database

We employed different levels of data curation and investigated their impact on model performance. The lowest level of curation consisted of careful treatment of duplicates, preprocessing of chemical structures, and excluding substances with concentrations in the influent below the limits of quantification (LOQ) (even if they were found in effluent samples). When the effluent concentration was below the LOQ, we still used the measured value to calculate breakthrough, but we systematically evaluated the impact of using these more uncertain values in the model, as described in the ‘Additional Curation’ section and detailed further in SI Section S11.

#### 2.3.1 The Role of WWTP Technology

WWTPs used in this study all employ conventional activated sludge processes. Of the 44 plants, 40 include a nitrifying-denitrifying step, which we refer to as nitrogen (N)-eliminating plants whereas the remaining 4 plants were only C-eliminating. The nitrifying-denitrifying step is known to significantly impact the removal of certain substances,^7^ raising concerns about how combining data from these two types of plants would affect model performance. We observed clear differences and therefore decided to restrict our model to data from N-eliminating plants, resulting in a more homogeneous data set. Thus, we do not include plant-specific parameters but rather aim for models that predict a rough expected breakthrough within typical conditions of this treatment technology. A deeper discussion is presented in the SI Section S5.

#### 2.3.2 Additional Curation

We systematically tested five additional curation criteria that could influence prediction quality. These criteria are (I) exclude substances for which breakthrough values could be calculated for less than three WWTPs, because there is less confidence in whether these values are representative of a wider range of treatment plants; (II) exclude substances with breakthrough values exceeding 120%, because these values could be the result of analytical errors or formation during treatment (i.e., transformation products); (III) exclude substances with high variability in breakthrough values across plants, because we assume it is more challenging to establish a structure–activity relationship for these substances as breakthrough seems to be strongly affected by subtle changes in treatment conditions; (IV) exclude entries with effluent values below the limit of quantification (LOQ); (V) exclude highly sorbing or highly volatile substances because we expect that the majority of substances in this study are removed via biodegradation so substances mainly removed by other mechanisms could introduce conflicting information in the models. For the latter purpose, we calculated organic carbon–water partition coefficients (K_OC_) and Henry’s law constants (H) of all substances using OPERA 2.9 (github.com/kmansouri/OPERA) and selected thresholds for exclusion of >4000 L/kg and >10^–5^ atm-m^3^/mol, respectively.

#### 2.3.3 Monitoring Campaigns

To ensure that measurements from different monitoring campaigns can be combined into one representative breakthrough value per substance, we tested for batch effects on a subset of 17 compounds that have more than three measurements per data set (SI Section S6). We found that the variability between substances is more important than the variability between data sets, justifying data pooling from the four monitoring campaigns.

### 2.4 Descriptors

We calculated several molecular representations, including molecular descriptors (PaDEL^24^ and Mordred^25^), and fingerprints (MACCS, Extended Connectivity Fingerprints (ECFP, radius = 2, length = 2048 bits; using RDKit^26^), and RDKit Fingerprints). Additionally, we created a novel fingerprint (ePFP) that indicates the presence of functional groups relevant for biodegradation. ePFP one-hot encodes triggered biotransformation rules from enviPath (i.e., a prediction system for microbial transformations^27^), which have been designed by experts and have been extensively validated:^28–31^ If the encoded functional group of the rule matches a molecule of interest, then the fingerprint value for this rule is set to 1, indicating that enzymatic transformation at this functional group might take place. Further details about the descriptors are provided in the SI Section S7.

### 2.5 Modeling

The target variable for model development was logB, the log-transformed breakthrough (B). As regressors we tested five linear models (Multiple Linear Regression (MLR), Ridge Regressor, Kernel Ridge Regressor (KR), Stochastic Gradient Descent Regressor (SGD), and Linear Support Vector Regressor (LSVR)), three ensemble regressors (Random Forest (RF) Gradient Boosting Regressor (GBR) and AdaBoost (AB)), a Decision Tree Regressor (DT), a Multilayer Perceptron (MLP; a type of neural network), a Support Vector Regressor with a radial basis function kernel (SVR), and a K-Nearest Neighbors Regressor (KNN). These algorithms cover a wide range of robust linear and nonlinear methods frequently used in QSAR modeling.

All regressors were evaluated using 5-fold nested cross validation (CV) as explained in the SI Section S8. This workflow ensures three key aspects: (i) the model never sees the test set during optimization, (ii) optimization is validated over a wide range of molecules to prevent overfitting, (iii) the performance of the model is determined using five different sets that cover the whole database. Model performance was calculated by averaging the coefficient of determination (R^2^) and the root mean squared error (RMSE) over the 5 test folds. Statistical analysis to investigate differences in performance among models was performed using Pingouin,^32^ a python library for statistical analyses. Additionally, we performed y-scrambling tests considering the full modeling workflow as recommended by Rücker et al.,^33^ that is, the same nested cross-validation, and repeating descriptor selection and hyperparameter optimization.

## 3 Results and Discussion

### 3.1 Systematic Evaluation of Data Sets

To understand the quality and contribution of the different data sets, we analyzed them in terms of measured breakthrough values and the chemical space covered (Figure 1). In Figure 1.a, the box for each data set contains all measurements from different wastewater treatment plants (WWTPs) for chemical substances unique to that data set, and *Multiple* refers to substances in at least two data sets. Notably, the SNF data set contributed several substances that frequently escaped treatment across different WWTPs. Since substances in the SNF data set were identified and quantified using reference standards, we also investigated potential systematic differences in breakthrough values due to variations in analytical methods. Figure S1 shows breakthrough values for substances shared between SNF and other data sets, using median values for comparison. The results demonstrate that, despite different analytical methods, similar breakthrough values were obtained for the same substances. Even when differences occurred, there was no consistent trend of under- or overestimation when using semiquantitative area-based methods to determine breakthrough. This finding has two key implications: (i) Consistent with recent studies,^18^ area-based removal calculations are sufficient for monitoring chemical substance breakthrough from WWTPs, and (ii) the target substances in the SNF data set show higher breakthrough, providing valuable examples of persistent structures to the model. The SNF data set focuses on structurally complex micropollutants such as pesticides and pharmaceuticals, while the other data sets include many molecules which are easier to degrade (e.g., organic acids, small peptides etc.).

**Figure 1:**
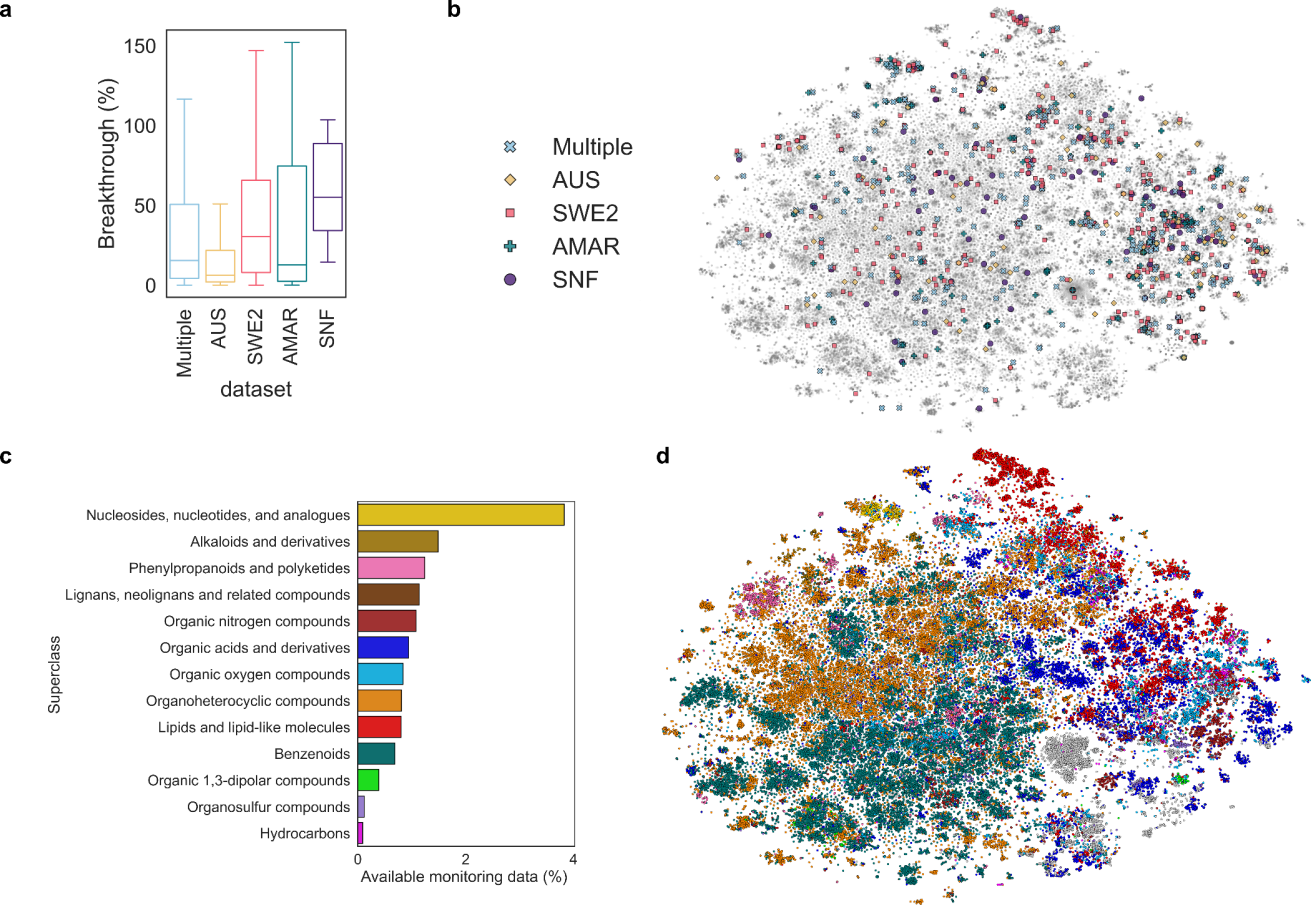
a) Calculated breakthroughs of substances unique to each data set; “Multiple” refers to breakthroughs of substances found in more than one data set. b) Two-dimensional representations of the chemical space covered by molecules included in this study in comparison with d) 134’000 marketed chemicals as described by von Borries et al.;^11^ the histogram c) shows the coverage (i.e., percent of substances covered in the monitoring campaigns relative to those included in the work of von Borries et al.^11^) in each class.

We also analyzed the substances in each data set in terms of their chemical structures. Figure 1b shows a two-dimensional representation of the chemical space of all the substances included in our data set. We used the t-distributed Stochastic Neighbor Embedding (t-SNE) to group similar molecules, where similarity is based on ECFPs. This is a common procedure to visualize the chemical space but often representations are not comparable. We therefore chose to reproduce the embedding of a recent publication^11^ as shown in Figure 1d, and mapped our substances within the same space of over 134’000 marketed chemicals. The figure shows that our data sets cover a wide chemical space with examples in all major classes of organic chemicals, without any clear indication of data set-specific clustering. Nonetheless, most classes are only sparsely covered. The adjacent histogram shows the coverage as a percentage relative to the number of “marketed chemicals” as presented by von Borries et al.^11^ The figure shows that there is currently experimental data available for roughly 1–3% substances of interest, highlighting the challenge of establishing a solid QSAR model but also the urgent need to develop such models.

### 3.2 Evaluation of Model Performance: Setting the Benchmark by Testing Different Regressor-Feature Pairs

We developed models to predict breakthrough values based on chemical features by exploring various combinations of regressors and feature sets. To ensure fair comparisons, each regressor-feature pair was evaluated using the same training and testing splits, following the nested CV method explained in the SI Section S8. The data used in this section was selected applying all the curation criteria described in Section 2.3 as these are the data with the highest confidence; this set contains 462 compounds. Preliminary analysis showed very poor performance for most regressors, particularly the linear regressor using ordinary least-squares (MLR), highlighting the benefits of using ML algorithms to capture the complexity of this task (Figure S4). Next, we discuss the results obtained for the best regressors RF, GBR and SVR.

The performance on unseen data for the best regressor-descriptor pairs is summarized in Figure 2. With one exception, regressors have, on average, comparable performance. RF performs slightly better than the other regressors, regardless of the descriptors selected or the feature reduction methods. Further details on the statistical significance of these differences are given in the SI Section S10. For any given regressor, the differences in performance using different descriptors were also small but MACCS tend to perform better. Moreover, we found that ePFP was not effective for predicting breakthrough. This poor performance was unexpected as substructures included in the ePFP are known to trigger biological transformations.^34^ However, since these rules are specific to enzymatic transformations, many substances trigger only a small subset of rules. Among the 1,037 substances with valid breakthrough values, only 577 had unique fingerprints. In comparison, the number of unique fingerprints using MACCS is significantly higher (909). We therefore attribute the difference in performance between ePFP and MACCS fingerprints to the higher incidence of collisions (i.e., samples with the same fingerprint) in ePFP and hence consider ePFP as insufficient for modeling.

**Figure 2:**
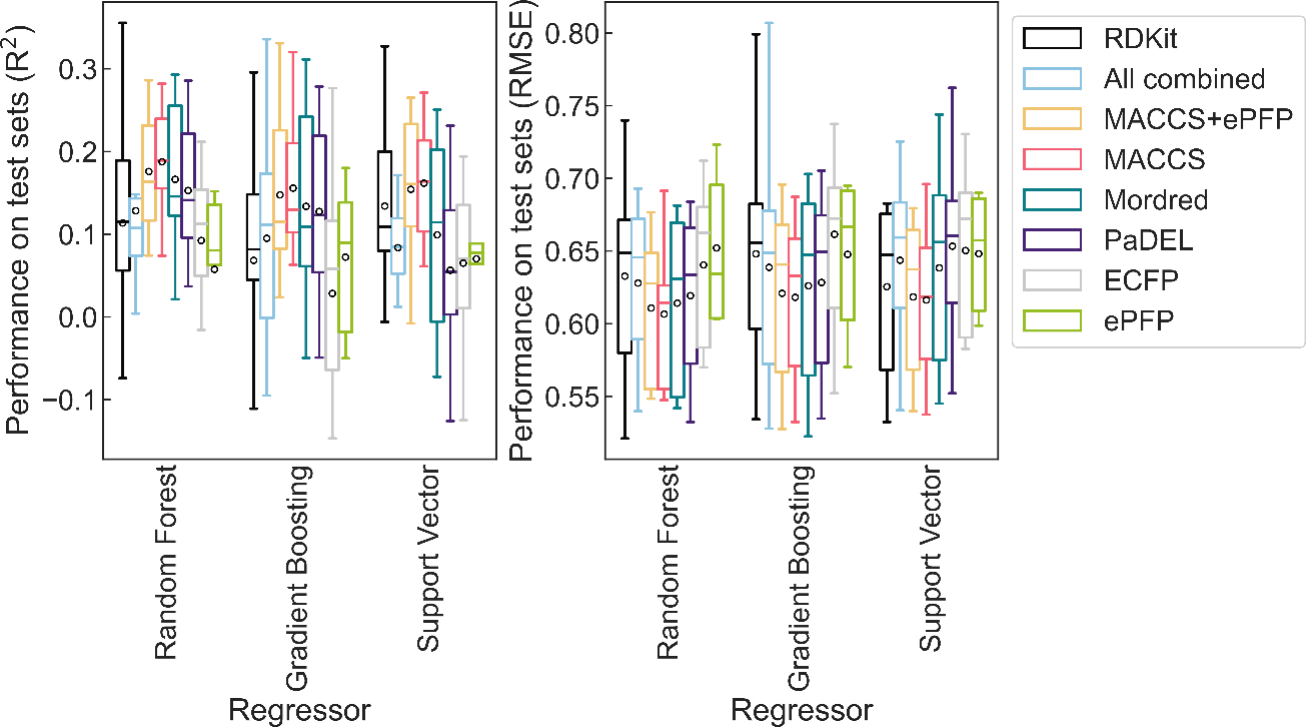
Performance of selected regressor-descriptors combinations on unseen data. Here the performance is evaluated on the subset of 462 chemicals that fulfill the strictest curation criteria. We cover the subset using 5-fold CV to maximize the amount of data available for training while ensuring performance is tested only on unseen data. The circle shows the mean performance of the 5-fold CV.

Our analyses revealed that similar performance may be obtained with different combinations of regressors and descriptors, yet there is a tendency of the RF-MACCS combination to do better, judging by the highest mean and median R^2^ and the lowest mean and median RMSE as shown in Figure 2. RF offers additional benefits: it performs well without feature selection, is inherently robust, has an out-of-bag (OOB) option to reduce overfitting, and, as an ensemble method, provides a measure of confidence in predictions. MACCS also have several advantages: they are easy to interpret because they refer to substructures with clear definitions, they are fast to calculate because calculations are limited to matching substructures, and training the models is also fast because MACCS result in very short fingerprints (i.e., > 10 times smaller than ECFPs). Interpretability is important for added confidence in the developed models and fast calculations are particularly important when screening very large numbers of chemicals. Given these advantages, we further optimized the MACCS-RF model.

### 3.3 Can We Improve the Models by Adding More Data during Training?

Since our models showed low predictive ability with unseen data (see Figure 2), we explored whether performance could be improved by additional training data. Specifically, we aimed to identify the quality criteria that defined the best training set. This is important for two reasons: (i) to optimize and select features using the most informative data, and (ii) to provide guidelines on which types of data should or should not be included in future expansions of the database.

The results of retraining the RF-MACCS model with different training sets covering all possible combinations of our additional curation criteria are shown in the SI Figure S6 in Section S11. The models are tested on unseen subsets (size = 92) of the data with the highest confidence (size = 462) to discern whether additional data with a higher uncertainty improves model performance or rather introduces noise. Moreover, different criteria also mean a different training size, which is also expected to have an important effect on performance. The best performances were achieved using all curation criteria (369 compounds) and combination I + III (856 compounds), that is, the combination of only compounds for which data from at least three WWTPs were available and only those for which the variability across plants was low (i.e., standard deviation in log units <0.7). Figure S5 shows the distribution of standard deviations in the complete data set and illustrates how, using this threshold, a large portion of variability is removed without losing many substances; nonetheless, variability across plants remains a major challenge in model development The systematic analysis of the impact of different criteria on model performance suggests that excluding entries below the LOQ (i.e., criterion IV) is not particularly beneficial, that is, highly uncertain values can still be informative and should be considered in future model development. We opted for combination (I + III) for further model development because it covers a larger chemical space leading to a broader applicability domain, and because restricting the domain of applicability does not compensate the small gain in performance.

### 3.4 Performance of the Final Model: Optimization and Comparison to Widely Used Regulatory Models

#### 3.4.1 Optimization

Models were finally optimized by 5-fold CV using the subset that is obtained upon using curation criteria I + III as explained in Section 3.3. We performed a randomized search over a large range of hyperparameter combinations and later a grid search over a smaller range close to the best combination found in the random search. When performance was the same, we gave priority to simpler models (i.e., smaller number of trees, smaller depth and larger number of minimum samples for a split) to reduce the risk of overfitting. Further details are provided in the SI Section S12 and Figure S7. As previously described by Sheridan et al.,^35^ RF models often overestimate low values and underestimate high values. We addressed this systematic bias following Sheridan’s method by applying a linear regression model to adjust the RF’s raw predictions. In this approach, the linear model is fitted on training data only and uses the relationship between RF predictions and actual breakthrough values from the training set to produce adjusted predictions. When reporting the performance of our optimized model, we refer to this adjusted prediction rather than the raw RF output. It is important to note that the performance of this fully optimized model is still rather poor, with R^2^ = 0.22 and RMSE = 0.68 as average performance on unseen data.

#### 3.4.2 Definition of Applicability Domain (AD)

As the final step to characterize our model, we aimed to define its domain of applicability. There is no established consensus on how AD should be defined but most approaches use either similarity metrics or ensemble agreement metrics.^35–39^ Among similarity metrics, the Tanimoto Similarity Index is widely used and has proven effective.^40,41^ This similarity measure is calculated pairwise; it can be defined as the similarity to the most similar molecule (i.e., *SimilarityNearest*) in the training set or as the average similarity to the five nearest molecules in the training set (i.e., *SimilarityNearest5*). A threshold is typically applied to determine whether a molecule falls within the model’s domain of applicability. In our case, we tested both *SimilarityNearest* and *SimilarityNearest5*. Rather than introducing a threshold, we investigated whether similarity to the training set correlated with improved predictions by ranking our predictions based on similarity and recalculating the RMSE as we progressively excluded a fraction of the set with the lowest similarity. We evaluated the RMSE of 18 subsets, ranging from all data to the top 10% most similar data in 5% increments. Figure S8 in the SI Section S13, shows that, when SimilarityNearest is used, RMSE decreases from 0.67 to 0.60 after removing 75% of most dissimilar molecules. This suggests that, in our case, SimilarityNearest alone is not a useful metric for identifying good predictions.

We observed better performance when using *SimilarityNearest5*, but it also required removing 70% of most dissimilar molecules to achieve RMSE below 0.60. These results indicate that, if we were to apply a similarity metric, an arbitrary threshold would lead to unreliable results and an optimized threshold (e.g., only top 25% most similar) would lead to RMSE below 0.6 but, at the same time, a very limited domain of applicability.

We therefore repeated the same analysis, but instead of ranking based on similarity, we ranked predictions by the standard deviation of the individual tree predictions in the ensemble (i.e., *TreeSD*). Here, we observed a consistent decrease in RMSE and increase in R^2^ as we removed predictions with the largest standard deviations, indicating that predictions were more accurate when the trees agreed more closely. For the top 25% predictions, the R^2^ is above 0.4 and RMSE below 0.5. Overall, we found that standard deviation in the predictions of individual trees serves as a strong indicator of confidence in predictions (further details in Section S13). To further contextualize these metrics, we simulated what would be the performance of a model that always predicts values within the observed experimental variability (see Section S13 for a more detailed explanation and simulation results shown in Figure S10). In that case, the resulting mean R^2^ would be 0.73 and the median RMSE 0.15.

#### 3.4.3 Interpretation of Final Model

Model performance on unseen data was lower than expected so understanding the model’s decisions is important for confidence in predictions. Moreover, OECD Principle 5 for QSAR models calls for a mechanistic association between the descriptors used and the end point, when possible.^42^ This association is essential when considering ML models for their use in regulatory contexts. Thus, we first calculated feature importance across the different folds in 5-fold CV; and retrained the model using only those most important features. Then we calculated the SHAP (SHapley Additive exPlanations) values for the fitted values (i.e., model “predictions” on the training set) to better understand the decisions taken by the model for molecules with a known breakthrough (Figure 3).

**Figure 3:**
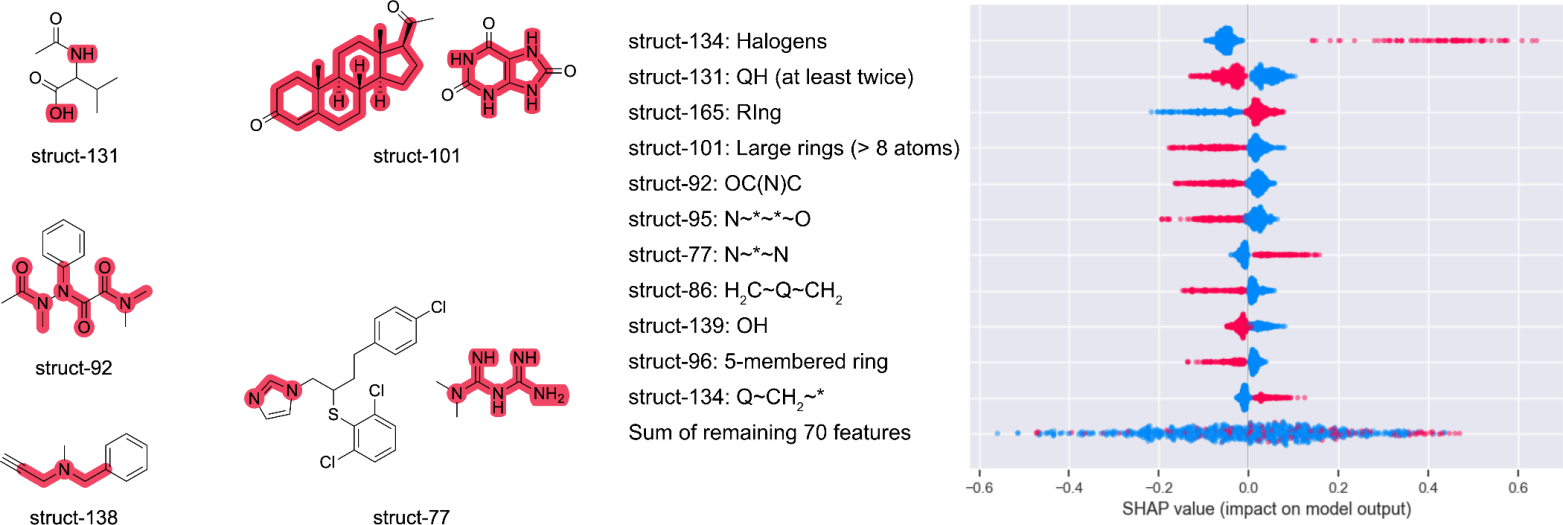
Shapley additive values of the substructures with the largest impact on model. Each circle represents a prediction, the color represents whether the substructure was present (red) or not (blue) in the structure of the predicted molecule. For example, the presence of struct-134 (i.e., halogens) always contributed to larger breakthroughs. The opposite is observed for struct-139 (i.e., −OH) where its presence always contributed to smaller breakthroughs. For substructures for which their definition is not evident, example molecules are given with the substructures highlighted in red.

Struct-134 (i.e., presence of -F, -Cl, -Br, -I) is both the most important feature during cross validation and the substructure with the largest impact on predictions when present. Figure 3 shows that the presence of a halogen is linked to larger breakthrough values, which is in line with current understanding of biodegradability.^43,44^ The absence of halogens (blue circles in struct-134 row) only moderately contributes to lower breakthroughs. A similar tendency is observed for struct-165, which refers to the presence of rings (both aromatic or aliphatic). If a ring structure is present, the model assigns larger breakthroughs, although to a lesser extent than if halogens are present. And differently from the halogens, the absence of rings highly contributes to lower breakthroughs, which can be observed from the large negative values of the blue circles for struct-165. Two more substructures have a similar tendency (i.e., higher breakthrough when present), i.e., struct-77, which refers to the pattern N∼*∼N where N is nitrogen, (*) is any atom and (∼) is any bond, and struct-138, which refers to the pattern Q ∼ CH_2_∼* where Q represents any atom which is not C or H. Struct-77 in many cases encodes the presence of imidazole, which is common in many pharmaceuticals (e.g., antifungals such as butoconazole) and is a moiety that is difficult to degrade. Guanidine-like substructures would also match struct-77 and these are often found in pharmaceuticals (e.g., metformin, a treatment against diabetes). Struct-138 is more general, note that both Q and (*) could be nitrogen atoms too but the central atom must be a CH_2_ which excludes imidazole, guanidine, carboxylic acids and amides. Common examples of compounds that contain struct-138 are tertiary amines.

Struct-131 normally matched terminal N and O atoms, like alcohols, carboxylic acids and primary amines which are expected to have low breakthroughs. Presence of struct-101 (8-membered rings or larger where adjacent rings are counted as a single ring) contributing to smaller breakthroughs is observed and rather counterintuitive. Examples of compounds that match struct-101 and have low observed breakthroughs include compounds with guanine-like substructures. Intuitively these aromatic rings are not expected to be easily biotransformed but there are plenty of naturally occurring substances with these substructures such as nucleotides and nucleosides. Differently, smaller aromatic rings (e.g., 6-membered rings such as phenols) which would encode struct-165 but not struct-101 are xenobiotic and presented larger breakthroughs, and this explains the tendency of struct-101 as a driver of lower breakthroughs. Finally, struct-139, which refers to the presence of −OH, is also selected as an important predictor and its presence always leads to smaller breakthroughs as expected.

#### 3.4.4 Benchmarking Model Performance

Next, we compared the predictions of the optimized model with those of the STPWIN tool from the EPI Suite.^45^ STPWIN is the only tool that allows predicting removal rates for a batch of chemicals based on chemical structure. There are other models with related endpoints (e.g., biodegradability classification tasks) which are discussed in SI Section S1 but direct comparison with these models was not possible. The agreement between the monitoring data and predictions of both our model and STPWIN is shown in Figure 4a. The RMSE for our predictions was 0.62, compared to 0.92 for STPWIN predictions. The R^2^ was 0.22 for our model and −0.70 for STPWIN. Recent assessment of the quality of predictions of STPWIN is missing, however, previous studies have reported prediction errors within 1–2 log units of magnitude for similar tools, such as SimpleTreat.^6,46^

**Figure 4:**
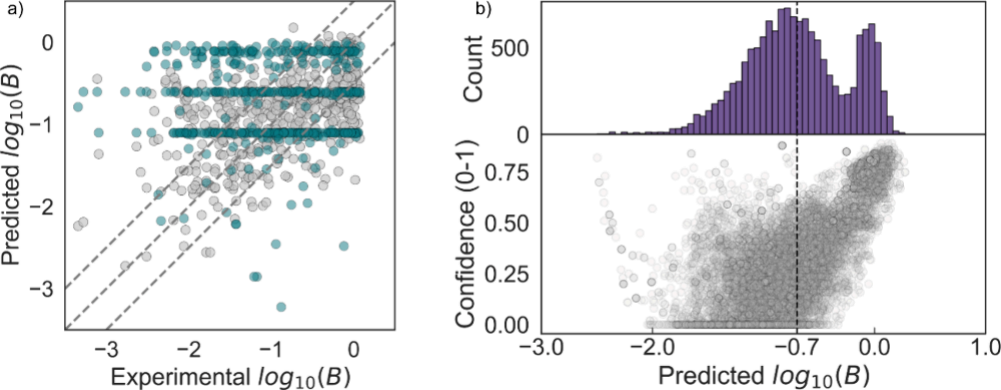
a) Predictions of this study (gray) in comparison with predictions using STPWIN (green) for 492 chemicals. To maximize the training data while ensuring that we test on unseen data, we retrained and tested the model 5 times so that in each run the model predicts unseen data; for simplicity, the 492 predictions are shown together. b) Predicted breakthrough for 14’000 unique organic chemical substances under REACH and the relationship between predicted breakthrough and model confidence.

We believe that the chosen test set of chemicals is particularly challenging for process-based models such as STPWIN and SimpleTreat, as most molecules in this subset are removed primarily via biotransformation, a complex mechanism that remains difficult to predict.^15^ Both STPWIN and SimpleTreat rely on physicochemical properties to estimate removal by various mechanisms, ultimately combining these individual predictions into a total removal value.^5^ STPWIN in particular outputs values for each individual mechanism, enabling us to calculate the fraction attributed to biodegradation. As expected, nearly all predictions rely heavily on biodegradation, indicating that prediction accuracy depends largely on the accuracy of the primary biotransformation rate constant (see SI Figure S11 and further discussion in SI Section S14). Similarly, Lautz et al.^46^ observed that errors were 10 times higher when using biotransformation rate constants predicted by BIOWIN in comparison to using measured rate constants. Their study also confirmed that using plant-specific reactor parameters did not improve predictions significantly compared to simply using default values, which also highlights the dominant weight of biotransformation rate constants in the prediction errors. Our modeling approach, which is purely data-driven but trained on a large set of actual WWTP monitoring data, performs better at predicting removals across a large range of compounds, despite the fact that it does not explicitly account for different removal processes. Nonetheless, models that encode operational conditions are likely to achieve better accuracies for specific plants and are highly desirable in the context of treatment design and operation, a notable example being the work of Zhang et al.^47^ We conclude that our model is an important alternative for predicting removal in WWTPs, particularly for substances that are mainly removed by biotransformation, where STPWIN and SimpleTreat are likely to fail as they need to rely on predictions by BIOWIN.

### 3.5 Applicability of the Model and Outlook

To illustrate the utility of our model, we predicted breakthrough for over 14’000 chemicals registered under REACH. This list of unique organic chemicals registered under REACH was compiled and curated by Arp & Hale.^12^ In Figure 4b, we present the distribution of predicted values along with the confidence in the predictions. Notably, 50% of chemicals are predicted to be well removed (i.e., log B < −0.7, B < 20%), yet most of them (i.e., 88%) with a low confidence (i.e., < 0.4). Interestingly, there is an obvious tendency for larger breakthroughs to be predicted with higher confidence. Based on the SHAP analysis and inspection of the structures predicted to have larger breakthroughs with a high confidence, we conclude that the model most likely correctly identifies recalcitrant substructures (e.g., structures containing halogens) and correctly assigns large breakthrough values. However, the sole absence of these recalcitrant substructures is obviously not enough to confidently assign a low breakthrough. In many cases, this may not be a systematic error of the model but rather a reflection of the variability in actual removal of these substances. From a precautionary perspective, the many predictions of low breakthrough with low confidence are problematic since there is a high likelihood that the model predicts many chemicals to be well removed when, in fact, they are not. Future efforts to confidently apply this and similar models for regulatory purposes and for SSbD should therefore focus on improving this aspect. For instance, substances with predicted low breakthrough and low prediction confidence could be prioritized for experimental testing in activated sludge assays and the results fed back into model development. Finally, the few molecules predicted to have a low breakthrough with high confidence are mostly carboxylic acids, alcohols, ethers, and guanine-like metabolites, which aligns well with current understanding of biodegradability.

Our models learn from increasingly available, semiquantitative monitoring data from WWTPs and are able to predict an expected breakthrough for a highly diverse set of organic molecules. These predictions proved more reliable than existing process-based models that are widely used in EU and US regulatory contexts, in cases where removal is mostly driven by biodegradation and especially for molecules where no experimental biotransformation kinetic data are available. This suggests that our model could be an important and novel contribution to the toolbox of *in silico* models used for alternatives assessment, when evaluating new molecules in industrial research and development, or even for exposure modeling in a risk assessment context. We have here established a benchmark model, which is publicly available along with the training data and the scripts necessary to reproduce the data curation process and retrain the models as new data become available (github.com/FennerLabs/pepper; web application: pepper-app.streamlit.app). Data availability has been one of the major bottlenecks to develop models in environmental science and it is a critical requirement to make full use of new developments in artificial intelligence. Therefore, we anticipate that this benchmark and the transparently curated data set that we provide will facilitate further developments in the field.

## Supplementary Material



## Notes

A brief description of related models, further analysis of the monitoring campaigns and the impacts of their differences for model development and performance, further details of statistical methods for model development and validation, an extended discussion on confidence metrics, and further details on the comparison of the performance of our models with respect to relevant existing tools (PDF)

## References

[1] Coll C., Fenner K., Screpanti C. (2023). Early Assessment of Biodegradability
of Small Molecules to Support the Chemical Design in Agro & Pharma
R&D. CHIMIA.

[2] Leder C., Suk M., Lorenz S., Rastogi T., Peifer C., Kietzmann M., Jonas D., Buck M., Pahl A., Kümmerer K. (2021). Reducing Environmental
Pollution by Antibiotics through Design for Environmental Degradation. ACS Sustain. Chem. Eng..

[3] Zumstein M. T., Fenner K. (2021). Towards More Sustainable
Peptide- Based Antibiotics:
Stable in Human Blood, Enzymatically Hydrolyzed in Wastewater?. CHIMIA.

[4] United States Environmental Protection Agency EPI SuiteTM-Estimation Program Interface. https://www.epa.gov/tsca-screening-tools/epi-suitetm-estimation-program-interface (accessed 2024–11–04).

[5] Struijs, J. SimpleTreat 4.0: A Model to Predict the Fate and Emission of Chemicals in Wastewater Treatment Plants; RIVM 601353005; Netherlands National Institute of Public Health and the Environment: 2014, p 65. https://www.rivm.nl/bibliotheek/rapporten/601353005.pdf.

[6] Comber S., Gardner M., Sörme P., Ellor B. (2019). The Removal of Pharmaceuticals
during Wastewater Treatment: Can It Be Predicted Accurately?. Sci. Total Environ..

[7] Jelic A., Gros M., Ginebreda A., Cespedes-Sánchez R., Ventura F., Petrovic M., Barcelo D. (2011). Occurrence, Partition
and Removal of Pharmaceuticals in Sewage Water and Sludge during Wastewater
Treatment. Water Res..

[8] Ortiz
de García S., Pinto Pinto G., García Encina P., Irusta Mata R. (2013). Consumption and Occurrence of Pharmaceutical and Personal
Care Products in the Aquatic Environment in Spain. Sci. Total Environ..

[9] Franco A., Struijs J., Gouin T., Price O. R. (2013). Evolution of the
Sewage Treatment Plant Model SimpleTreat: Use of Realistic Biodegradability
Tests in Probabilistic Model Simulations. Integr.
Environ. Assess. Manag..

[10] Kim H.-J., Lee H.-J., Lee D. S., Kwon J.-H. (2009). Modeling the Fate
of Priority Pharmaceuticals in Korea in a Conventional Sewage Treatment
Plant. Environ. Eng. Res..

[11] von
Borries K., Holmquist H., Kosnik M., Beckwith K. V., Jolliet O., Goodman J. M., Fantke P. (2023). Potential for Machine
Learning to Address Data Gaps in Human Toxicity and Ecotoxicity Characterization. Environ. Sci. Technol..

[12] Arp H. P. H., Hale S. E. (2022). Assessing the Persistence
and Mobility of Organic Substances
to Protect Freshwater Resources. ACS Environ.
Au.

[13] Posthumus R., Traas T. P., Peijnenburg W. J. G.
M., Hulzebos E. M. (2005). External
Validation of EPIWIN Biodegradation Models. SAR QSAR Environ. Res..

[14] Acharya K., Werner D., Dolfing J., Barycki M., Meynet P., Mrozik W., Komolafe O., Puzyn T., Davenport R. J. (2019). A Quantitative
Structure-Biodegradation Relationship (QSBR) Approach to Predict Biodegradation
Rates of Aromatic Chemicals. Water Res..

[15] Jiang S., Liang Y., Shi S., Wu C., Shi Z. (2023). Improving
Predictions and Understanding of Primary and Ultimate Biodegradation
Rates with Machine Learning Models. Sci. Total
Environ..

[16] Wang L., Lei Z., Yun S., Yang X., Chen R. (2024). Quantitative Structure-Biotransformation
Relationships of Organic Micropollutants in Aerobic and Anaerobic
Wastewater Treatments. Sci. Total Environ..

[17] Nolte T. M., Chen G., van Schayk C. S., Pinto-Gil K., Hendriks A. J., Peijnenburg W. J. G.
M., Ragas A. M. J. (2020). Disentanglement
of the Chemical, Physical, and Biological Processes Aids the Development
of Quantitative Structure-Biodegradation Relationships for Aerobic
Wastewater Treatment. Sci. Total Environ..

[18] Chirico N., McLachlan M. S., Li Z., Papa E. (2024). In Silico Approaches
for the Prediction of the Breakthrough of Organic Contaminants in
Wastewater Treatment Plants. Environ. Sci.:
Processes Impacts.

[19] Soares T. A., Nunes-Alves A., Mazzolari A., Ruggiu F., Wei G.-W., Merz K. (2022). The (Re)-Evolution
of Quantitative Structure-Activity Relationship
(QSAR) Studies Propelled by the Surge of Machine Learning Methods. J. Chem. Inf. Model..

[20] Siemers F. M., Feldmann C., Bajorath J. (2022). Minimal Data
Requirements for Accurate
Compound Activity Prediction Using Machine Learning Methods of Different
Complexity. Cell Rep. Phys. Sci..

[21] Wang Q. L., Apul O. G., Xuan P., Luo F., Karanfil T. (2013). Development
of a 3D QSPR Model for Adsorption of Aromatic Compounds by Carbon
Nanotubes: Comparison of Multiple Linear Regression, Artificial Neural
Network and Support Vector Machine. RSC Adv..

[22] Huang K., Zhang H. (2022). Classification and
Regression Machine Learning Models for Predicting
Aerobic Ready and Inherent Biodegradation of Organic Chemicals in
Water. Environ. Sci. Technol..

[23] Schymanski E. L., Jeon J., Gulde R., Fenner K., Ruff M., Singer H. P., Hollender J. (2014). Identifying Small Molecules via High
Resolution Mass Spectrometry: Communicating Confidence. Environ. Sci. Technol..

[24] Yap C. W. (2011). PaDEL-Descriptor:
An Open Source Software to Calculate Molecular Descriptors and Fingerprints. J. Comput. Chem..

[25] Moriwaki H., Tian Y.-S., Kawashita N., Takagi T. (2018). Mordred: A Molecular
Descriptor Calculator. J. Cheminformatics.

[26] Python API Reference — The RDKit 2024.03.4 documentation. http://rdkit.org/docs/api-docs.html (accessed 2024–07–01).

[27] Hafner J., Lorsbach T., Schmidt S., Brydon L., Dost K., Zhang K., Fenner K., Wicker J. (2024). Advancements
in Biotransformation
Pathway Prediction: Enhancements, Datasets, and Novel Functionalities
in enviPath. J. Cheminformatics.

[28] Trostel L., Coll C., Fenner K., Hafner J. (2023). Combining Predictive
and Analytical Methods to Elucidate Pharmaceutical Biotransformation
in Activated Sludge. Environ. Sci. Process.
Impacts.

[29] Schmid, E. ; Fenner, K. enviLink: A Database Linking Contaminant Biotransformation Rules to Enzyme Classes in Support of Functional Association Mining. bioRxiv Bioinformatics May 21, 2021. 10.1101/2021.05.20.442588.

[30] Wicker J., Fenner K., Ellis L., Wackett L., Kramer S. (2010). Predicting
Biodegradation Products and Pathways: A Hybrid Knowledge- and Machine
Learning-Based Approach. Bioinformatics.

[31] Hou B. K., Ellis L. B. M., Wackett L. P. (2004). Encoding Microbial Metabolic Logic:
Predicting Biodegradation. J. Ind. Microbiol.
Biotechnol..

[32] Vallat R. (2018). Pingouin:
Statistics in Python. J. Open Source Softw..

[33] Rücker C., Rücker G., Meringer M. (2007). Y-Randomization and Its Variants
in QSPR/QSAR. J. Chem. Inf. Model..

[34] Ellis L. B.
M., Gao J., Fenner K., Wackett L. P. (2008). The University of
Minnesota Pathway Prediction System: Predicting Metabolic Logic. Nucleic Acids Res..

[35] Sheridan R. P. (2013). Using Random
Forest To Model the Domain Applicability of Another Random Forest
Model. J. Chem. Inf. Model..

[36] Liu R., Wallqvist A. (2019). Molecular
Similarity-Based Domain Applicability Metric
Efficiently Identifies Out-of-Domain Compounds. J. Chem. Inf. Model..

[37] Wang Z., Chen J., Hong H. (2020). Applicability
Domains Enhance Application
of PPARγ Agonist Classifiers Trained by Drug-like Compounds
to Environmental Chemicals. Chem. Res. Toxicol..

[38] Sheridan R. P. (2015). The Relative
Importance of Domain Applicability Metrics for Estimating Prediction
Errors in QSAR Varies with Training Set Diversity. J. Chem. Inf. Model..

[39] Korolev V., Mitrofanov A., Korotcov A., Tkachenko V. (2020). Graph Convolutional
Neural Networks as “General-Purpose” Property Predictors:
The Universality and Limits of Applicability. J. Chem. Inf. Model..

[40] Reymond J.-L. (2022). Molecular
Similarity for Drug Discovery, Target Prediction and Chemical Space
Visualization. CHIMIA.

[41] Gini G. (2020). The QSAR Similarity
Principle in the Deep Learning Era: Confirmation or Revision?. Found. Chem..

[42] OECD Guidance Document on the Validation of (Quantitative) Structure-Activity Relationship [(Q)SAR] Models; OECD Series on Testing and Assessment, No. 69; OECD Publishing: Paris, 2014. https://www.oecd.org/en/publications/guidance-document-on-the-validation-of-quantitative-structure-activity-relationship-q-sar-models_9789264085442-en.html (accessed 2025–09–06).

[43] Boethling R. S., Howard P. H., Meylan W., Stiteler W., Beauman J., Tirado N. (1994). Group Contribution Method for Predicting Probability
and Rate of Aerobic Biodegradation. Environ.
Sci. Technol..

[44] Straub J. O., Le Roux J., Tedoldi D. (2023). Halogenation
of Pharmaceuticals Is
an Impediment to Ready Biodegradability. Water.

[45] van
Dijk J., Flerlage H., Beijer S., Slootweg J. C., van Wezel A. P. (2022). Safe and
Sustainable by Design: A Computer-Based Approach to Redesign Chemicals
for Reduced Environmental Hazards. Chemosphere.

[46] Lautz L. S., Struijs J., Nolte T. M., Breure A. M., van der
Grinten E., van de Meent D., van Zelm R. (2017). Evaluation of SimpleTreat
4.0: Simulations of Pharmaceutical Removal in Wastewater Treatment
Plant Facilities. Chemosphere.

[47] Zhang C., Yu Q., He Y., Wu G., Fan F., Xu K., Ren H., Geng J. (2025). Decoding Pharmaceutical
Removability in Full-Scale
Wastewater Treatment Plants via Machine Learning Model Integrating
Modified Electrophilicity Index. Water Res..

