# Supplementary material for: Predicting Micropollutant Removal in Wastewater Treatment Based on Molecular Structure: Benchmark Data and Models
Source: Environ Sci Technol. 2025 Oct 8;59(41):22020–8. doi: 10.1021/acs.est.5c09314 (PMC12550809; doi:10.1021/acs.est.5c09314)
Supplement: Supplementary file 1 [file es5c09314_si_001.pdf]

Supporting Information for:

Predicting Micropollutant Removal in Wastewater Treatment based on Molecular Structure – Benchmark Data and Models

J.A. Cordero[1*], *J. Hafner*[2], *M. S. McLachlan*[3], *H. Singer*[1], *K. Fenner*[1,2]

1. **Department of Environmental Chemistry, Swiss Federal Institute of Aquatic Science and Technology (Eawag)**
2. **Department of Chemistry, University of Zürich**
3. **Department of Environmental Science, University of Stockholm**

*Corresponding author: jose.cordero@eawag.ch

# of pages: 28

# of tables: 4

# of figures: 11

Table of Contents

List of Figures 2
List of Tables 3
1 Related models and tools 4
2 Detailed information of datasets 5
3 Information on python package PEPPER 8
4 Curation of the database 8
5 WWTP technology 9
6 Analysis of batch effects across monitoring campaigns 11
7 Descriptors 14
8 Nested cross validation 14
9 Preliminary models 15
10 Statistical Significance of the Differences in Performance for Different Regressors 17
11 Statistical Significance of the Differences in Performance when Training with Different subsets 17
12 Hyperparameter optimization & bias correction 21
13 Applicability domain & Confidence metric 23
14 Analysis of predictions using STPWIN 26
15 References 28

List of Figures

Figure S1.Scatter plot showing measured values for chemicals for which breakthroughs were determined using areas (Level 2) or quantified with standards (Level 1). The error bars in the scatterplot represent the standard deviation of the values across different WWTPs for a single compound. The dotted line delineates a difference in 0.5 log units. 7
Figure S2. Scatter plot showing measured breakthrough values as log10 (Breakthrough) for plants with nitrifying/denitrifying capabilities (N-eliminating) vs conventional sludge (C-eliminating). 10
Figure S3: **Top**: The distribution of logB by dataset for all 17 compounds with at least 3 measurements from every monitoring campaign. **Bottom left**: The distribution of logB for the 3 compounds with measurements available every WWTP. **Bottom right:** The distribution of the standard deviation of logB values by compound within datasets and for all datasets taken together. Only the 17 compounds with at least 3 measurements from every monitoring campaign were considered. 12

Figure S4. Performance of the different regressors tested. Multiple Linear Regression (MLR) using Ordinary Least Squares (OLS) was also tested but not included in the figure. The RMSE was above 10 and R^2 below -30. 16
Figure S5. Distribution of the standard deviation of breakthrough values (in log scale) of individual molecules across the whole dataset. Each count represents one molecule and the standard deviation is calculated across the whole dataset. 19
Figure S6. Performance using different data subsets for training when using additional curation criteria. The criteria are identified as follows: (I) Only data available in 3 WWTPs or more, (II) No substances with breakthrough above 120%, (III) No substances with large variability across WWTPs and (IV) only measurements above the limit of quantification (V) Remove volatile and highly sorbing substances. The number that follows the curation strategies is the number of chemicals that fulfill such criteria. 20
Figure S7. Initial randomized search and b) narrower grid search to select the best hyperparameters using 5-fold cross validation. The y-axis represents the mean scores of the cross validation for a given combination of hyperparameters and the x-axis represents the standard deviation of the scores among folds. We consider that a lower standard deviation is evidence of a more robust model, so we selected the hyperparameters that yielded the best score with the lowest standard deviation. 21
Figure S8. Model performance as a function of confidence in predictions using similarity metrics and ensemble prediction agreement metrics. Using the standard deviation in the predictions of the individual trees (TreeSD) is a good measure of confidence as it leads to lower errors. 23
Figure S9. Raw values that correspond with the ranked percentage values in the previous figure. This allows visualization of the actual thresholds and their expected performance. Note that the x-axis is reversed. The interpretation is that in general smaller TreeSD leads to lower error and higher R2 and the opposite for Similarity metrics where decreased similarity ends to result in worse performance (higher RMSE and lower R2). 24
Figure S10. Simulated maximum achievable performance by simulating a model that predicts values from the actual experimental values. Conceptually it illustrates a model with an accuracy that matches experimental variability. 25
Figure S11. Predictions STPWIN. Predictions of STPWIN. a) The color code represents the fraction of total removal attributed to biodegradation. b) Predictions for chemicals where biodegradation represents less than 40% of the total removal 27

List of Tables

Table S1 Selection of models and tools with a similar endpoint or context to this study 4
Table S2. Summary information on datasets and the wastewater treatment plants associated. 5
Table S3: 2-way ANOVA results investigating the effect of dataset, compound structure and their combination on the variance of logB. 13
Table S4. Set of optimal parameters for the best model. 23

1 Related models and tools

Table S1 shows some models and tools with a target endpoint similar to this study. STPWIN, SimpleTreat and Chirico et al. (2024) aim to predict removal of chemicals in wastewater treatment plants. Differently Nolte et al. (2020) aimed to build QSAR models to predict biodegradation rates using full-scale WWTP data. Zhang et al. (2025) used full-scale data to classify compounds based on their expected removability. Huang & Zhang notably uses a very large dataset but these are records from lab-scale experiments and not WWTP data; moreover, it is a classification model. Wang et al. (2024) did a regression a task but their objective was also not related to WWTPs. These models have some relationship with our study but none of them target the same endpoint with a similar strategy (i.e., machine learning supported quantitative structure-activity relationship models). We also include the review of Rios-Miguel et al. (2023), as it covers studies focusing on removal of micropollutants in WWTPs with special emphasis in microbial degradation.

Table S1 Selection of models and tools with a similar endpoint or context to this study

Tool name OR reference	Endpoint	Method	N substances	Performance
STPWIN	Removal WWTPs	Compartment-based	12	Summary statistics not provided
SimpleTreat	Removal WWTPs	Compartment-based	43 for testing	Effluent estimate within factor of 10 (according to Lautz et al. 2017)
Nolte et al. 2020	Biodegradation rates in WWTPs	QSAR-Linear regression	69	R2_validation = 0.70
Huang & Zhang. 2022	Readily biodegradable (classification)	QSAR-Machine Learning	~6000	85% accuracy
Wang et al. 2024	Biodegradation rate constants	QSAR-Linear regression	14	R2 > 0.5
Chirico et al. 2024	Breakthrough WWTPs	QSAR-Linear regression	70	MAE_test = 0.62
Zhang et al. 2025	Removability in WWTPs (Classification)	QSAR-Machine learning	80	0.81 accuracy
Rios-Miguel et al. 2023	Review: Microbial degradation in WWTPs*			

*The list is not exhaustive for biodegradation related endpoints; The review of Rios-Miguel presents a more comprehensive list

2 Detailed information of datasets

As explained in the main manuscript, the data used in this study is a compilation of 4 smaller datasets, i.e., AMAR, AUS, SNF and SWE2. Table S2 summarizes information about the datasets. The AMAR dataset was collected as part of a thesis supervised by the co-author Michael MacLachlan (MM) and all the experimental details can be found online in this work[1]. For details on the AUS datasets please refer to this publication[2]. The SNF data was collected as part of a project funded by the Swiss National Foundation and supervised by co-author Kathrin Fenner, the experimental details are described in the thesis[3] and in the manuscript in preparation[4]. Finally, the SWE2 data have not been published but the methods are similar to those in the AMAR dataset, and the project was supervised by MM as well; a manuscript describing details of the SWE2 dataset is currently in preparation (co-authors: Yijing Li, Malte Posselt & Michael S. McLachlan).

Table S2. Summary information on datasets and the wastewater treatment plants associated.

Dataset	WWTP code	Country	Technology	Sampling points	Quantification	Identification level
AUS	S25	AUS	C-eliminating AS	inf/eff whole plant	semi-quantiatitive	level 2
	S2	AUS	AS-N/DN	inf/eff whole plant	semi-quantitative	level 2
	S5	AUS	AS-N/DN	inf/eff whole plant	semi-quantitative	level 2
	S11	AUS	AS-N/DN	inf/eff whole plant	semi-quantitative	level 2
	S24	AUS	AS-N/DN	inf/eff whole plant	semi-quantitative	level 2
	S40	AUS	AS-N/DN	inf/eff whole plant	semi-quantitative	level 2
AMAR	Enköping	SWE	C-eliminating AS	inf/effbiological treatment	semi-quantiatitive	level 2
	Katrineholm	SWE	AS-N/DN	inf/effbiological treatment	semi-quantitative	level 2
	Käppala	SWE	AS-N/DN	inf/effbiological treatment	semi-quantitative	level 2
	Luggage Point	AUS	AS-N/DN	inf/effbiological treatment	semi-quantitative	level 2

	Neugut	CH	AS-N/DN	inf/effbiological treatment	semi-quantitative	level 2
	Werdhölzli	CH	AS-N/DN	inf/effbiological treatment	semi-quantitative	level 2
SNF	Airolo	CH	C-eliminating AS	inf/eff whole plant	refrence standard	level 1
	Olivone	CH	C-eliminating AS	inf/eff whole plant	refrence standard	level 1
	Alt	CH	AS-N/DN	inf/eff whole plant	refrence standard	level 1
	Bir	CH	AS-N/DN	inf/eff whole plant	refrence standard	level 1
	Ehr	CH	AS-N/DN	inf/eff whole plant	refrence standard	level 1
	Kol	CH	AS-N/DN	inf/eff whole plant	refrence standard	level 1
SWE2	WWTP_1	SWE	AS-N/DN	inf/eff whole plant	semi-quantitative	level 2
	WWTP_2	SWE	AS-N/DN	inf/eff whole plant	semi-quantitative	level 2
	WWTP_3	SWE	AS-N/DN	inf/eff whole plant	semi-quantitative	level 2
	WWTP_4	SWE	AS-N/DN	inf/eff whole plant	semi-quantitative	level 2
	WWTP_5	SWE	AS-N/DN	inf/eff whole plant	semi-quantitative	level 2
	WWTP_6	SWE	AS-N/DN	inf/eff whole plant	semi-quantitative	level 2
	WWTP_7	SWE	AS-N/DN	inf/eff whole plant	semi-quantitative	level 2
	WWTP_8	SWE	AS-N/DN	inf/eff whole plant	semi-quantitative	level 2
	WWTP_9	SWE	AS-N/DN	inf/eff whole plant	semi-quantitative	level 2
	WWTP_10	SWE	AS-N/DN	inf/eff whole plant	semi-quantitative	level 2
	WWTP_11	SWE	AS-N/DN	inf/eff whole plant	semi-quantitative	level 2
	WWTP_12	SWE	AS-N/DN	inf/eff whole plant	semi-quantitative	level 2
	WWTP_13	SWE	AS-N/DN	inf/eff whole plant	semi-quantitative	level 2
	WWTP_14	SWE	AS-N/DN	inf/eff whole plant	semi-quantitative	level 2
	WWTP_15	SWE	AS-N/DN	inf/eff whole plant	semi-quantitative	level 2
	WWTP_16	SWE	AS-N/DN	inf/eff whole plant	semi-quantitative	level 2
	WWTP_17	SWE	AS-N/DN	inf/eff whole plant	semi-quantitative	level 2
	WWTP_18	SWE	AS-N/DN	inf/eff whole plant	semi-quantitative	level 2
	WWTP_19	SWE	AS-N/DN	inf/eff whole plant	semi-quantitative	level 2
	WWTP_20	SWE	AS-N/DN	inf/eff whole plant	semi-quantitative	level 2

WWTP_21	SWE	AS-N/DN	inf/eff whole plant	semi-quantitative	level 2
WWTP_22	SWE	AS-N/DN	inf/eff whole plant	semi-quantitative	level 2
WWTP_23	SWE	AS-N/DN	inf/eff whole plant	semi-quantitative	level 2
WWTP_24	SWE	AS-N/DN	inf/eff whole plant	semi-quantitative	level 2
WWTP_25	SWE	AS-N/DN	inf/eff whole plant	semi-quantitative	level 2
WWTP_26	SWE	AS-N/DN	inf/eff whole plant	semi-quantitative	level 2

Activated sludge with nitrification/denitrification (AS-N/DN)

The breakthrough values corresponding to data from AMAR, AUS and SWE2 datasets were calculated using the ratio of areas because absolute concentrations were not quantified. There is great benefit in using areas because it allows to dramatically increase the number of substances covered from 184 to 1153. A thorough analysis of the reliability of using areas rather than concentrations for determining breakthroughs has been conducted by McLachlan et al.[2]. Based

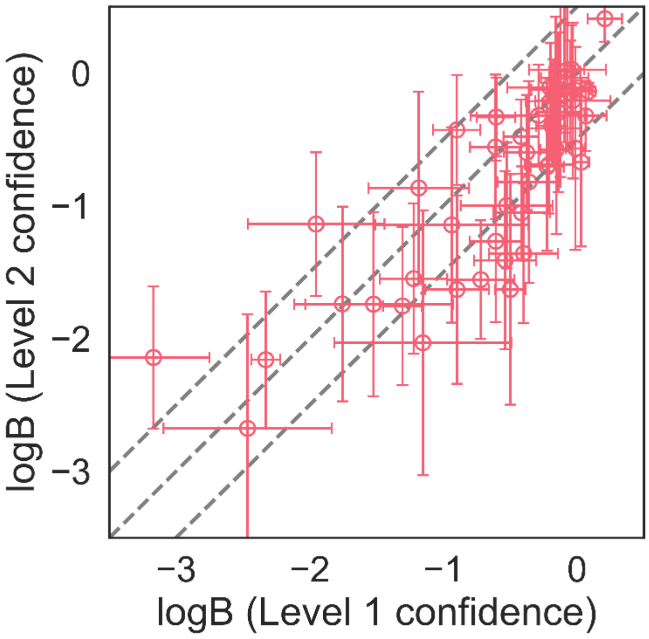

Figure S1.Scatter plot showing measured values for chemicals for which breakthroughs were determined using areas (Level 2) or quantified with standards (Level 1). The error bars in the scatterplot represent the standard deviation of the values across different WWTPs for a single compound. The dotted line delineates a difference in 0.5 log units.

on this evidence, we consider that the benefit of extending the chemical space outweighs the uncertainty associated with not using reference standards for quantification of absolute concentrations. Furthermore, we compared the measured values for all compounds for which data using areas and absolute concentrations were available. Figure S1 shows a good agreement between both values. The error bars represent the standard deviation for measurements across plants, which demonstrate that differences across plants are larger than differences in the measurements with different quantification methods. Finally, we did not observe any systematic bias, that is, differences among quantification methods are random.

3 Information on python package PEPPER

PEPPER (pypi.org/project/pepper-lab/) consists of three main modules: Data Structure, which includes methods for dataset curation (see Section 2.3); Descriptors, which generates molecular representations (see Section 2.4); and Modeling, which provides both in-house methods and scikit-learn wrappers for model development, including machine learning regressors, feature reduction, optimization, and evaluation techniques. Currently, this package supports a complete exploratory workflow to identify the optimal set of descriptors and regressors for a given dataset (see Section 2.5 for details). PEPPER also includes methods beyond the scope of this paper that will help users to model other environmentally relevant endpoints.

4 Curation of the database

For each substance in the datasets, the InChIKey and Canonical SMILES strings were retrieved from PubChem using PubChemPy. Substances with the same Canonical SMILES but different InChIKeys (e.g., stereoisomers) were treated as the same substance. Each substance may appear

only once per WWTP, so when duplicates were identified, the average breakthrough value was used to generate a single entry. Additionally, we manually inspected the HPLC retention times associated with these duplicates and excluded cases where retention times did not match. Each substance-plant pair was confirmed as unique before model development to prevent data leakage; note that entries for the same substance for different plants are expected. The complete curation process, including traceable annotations linking merged entries to the original data, is documented in PEPPER.

5 WWTP technology

We explored differences in the measured values for substances common to both plant types. Figure S2 shows the relationship between the median breakthrough values from C-eliminating plants and N-eliminating plants for the 149 substances with data from both technologies. We observed significant differences for many substances, which aligns with previous studies examining differences between these two treatment technologies.[5,6]

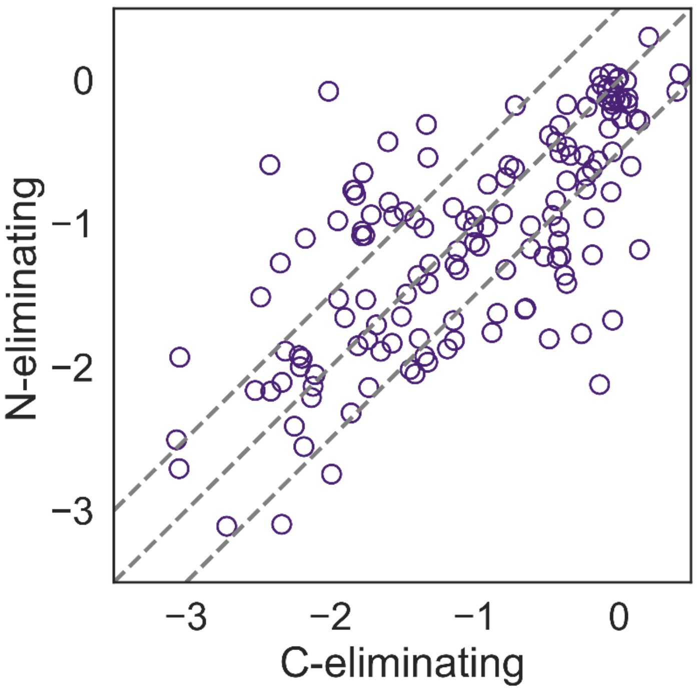

Figure S2. Scatter plot showing measured breakthrough values as log10 (Breakthrough) for plants with nitrifying/denitrifying capabilities (N-eliminating) vs conventional sludge (C-eliminating).

Although data from C-eliminating plants is available for many substances, it represents only 5% of the dataset in terms of individual records. Consequently, the values used for modeling typically align more closely with data from N-eliminating plants due to their dominant representation. This imbalance introduces two potential drawbacks: (i) combining data from both technologies could introduce conflicting information, suggesting that better performance might be achieved using only N-eliminating data; and (ii) this bias during training may lead to similarly biased predictions, making the model less suitable for predicting breakthroughs from C-eliminating plants.

Therefore, we developed our model relying on data from N-eliminating plants only and warn users that predictions better reflect expected behavior in plants with this technology. In the

future, as new data becomes available, we aim to explore strategies that allow users to adjust for these differences. We foresee two possible approaches: (i) encoding treatment conditions during training so that the model learns to predict for each plant type, and (ii) developing an additional model to adjust predictions from N-eliminating to C-eliminating conditions. While conceptually similar, these approaches involve different model architectures and would require additional data from C-eliminating plants, which falls outside the scope of this work. Therefore, we proceeded training models using data from N-eliminating plants only.

6 Analysis of batch effects across monitoring campaigns

To assess whether logB measurements from different monitoring campaigns for a same compound can be averaged into one representative logB value (i.e., median), we investigated how much variability in logB is due to the monitoring campaign. To do this, we first had to extract a subset of the data that allows us to quantify inter- and intra-group variability. We therefore extracted all compounds for which 3 or more measurements were available for all four monitoring campaigns, resulting in 774 measurements for 17 compounds. The results were plotted by compound, showing that for most compounds, the variability within monitoring campaigns is comparable to the variability between monitoring campaigns (Figure S*3*, top). Additionally, we had a closer look at the 3 compounds for which at least one measurement was available for every WWTP in the dataset (Figure S*3*, bottom left). The logB distribution of the paracetamol, DEET and sitagliptin nicely illustrate the three categories of compounds we want to distinguish: the easily and always biodegradable (paracetamol), the recalcitrant (sitagliptin), and the ones whose persistence is heavily influenced by WWTP conditions and other environmental factors. Finally, we looked at the standard deviation of logB by compound: For each of the 17 reference compounds, the standard deviation of logB was calculated for each dataset separately,

and also for all datasets taken together (Figure S3, bottom right). We can see that the amar_data has the highest average standard deviation (0.43), followed by swe2_data (0.38), aus_data (0.26), and snf_data (0.16). When all datasets are taken together, the mean standard deviation for a single compound is 0.40.

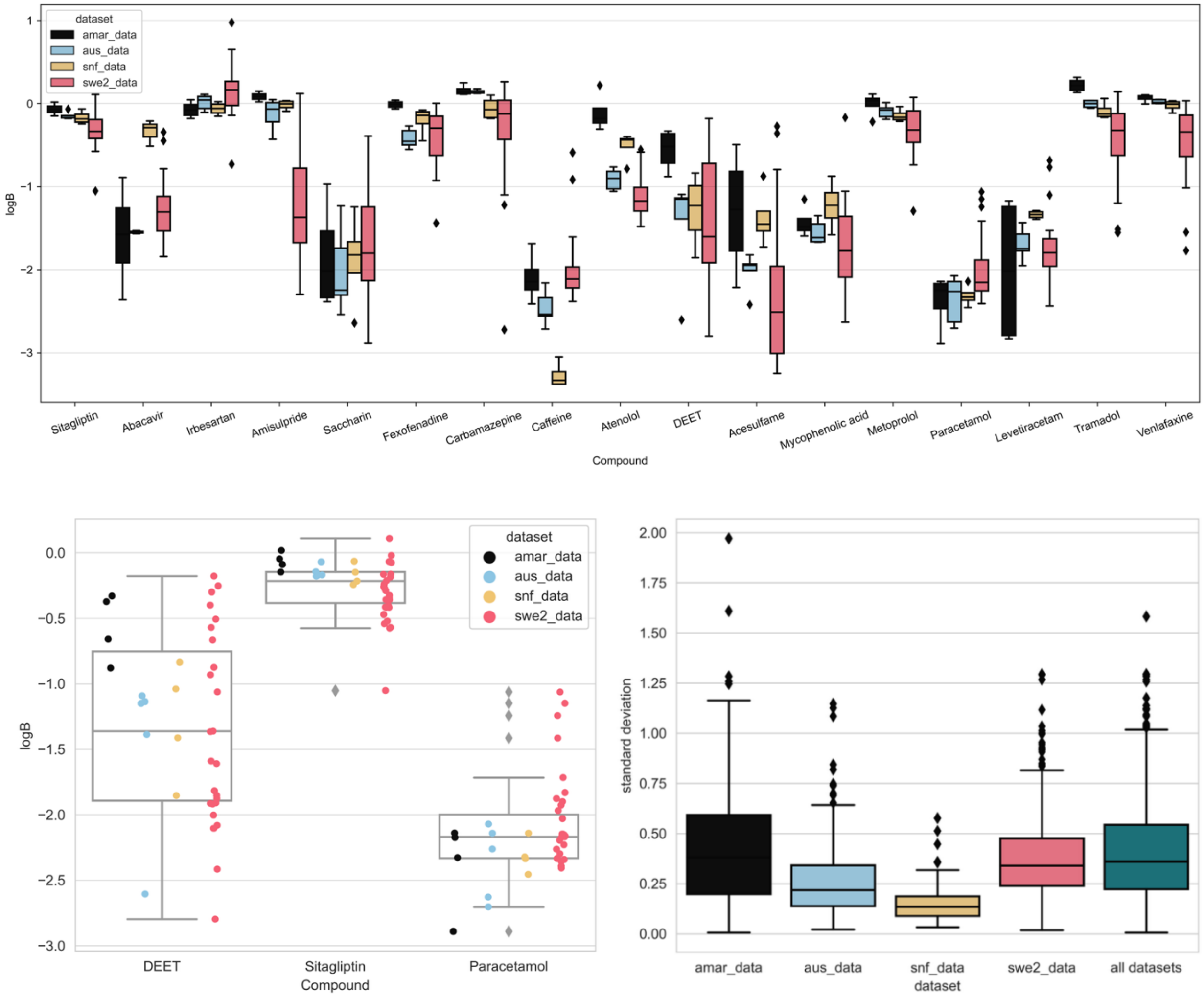

Figure S3: **Top**: The distribution of logB by dataset for all 17 compounds with at least 3 measurements from every monitoring campaign. **Bottom left**: The distribution of logB for the 3 compounds with measurements available every WWTP. **Bottom right:** The distribution of the standard deviation of logB values by compound within datasets and for all datasets taken

together. Only the 17 compounds with at least 3 measurements from every monitoring campaign were considered.

In addition to the visual assessment, a 2-way Analysis of Variance (ANOVA) was performed to quantify the variance that is due to (i) the monitoring campaign (‘dataset’), (ii) the compound structure, and (iii) the interaction effect of dataset x compound (Table S*3*).

Table S3: 2-way ANOVA results investigating the effect of dataset, compound structure and their combination on the variance of logB.

	**sum_sq**	**df**	**F**	**PR(>F)**
**C(dataset)**	7.497568	3	11.519221	2.52E-07
**C(compound)**	361.793599	16	104.223313	8.48E-152
**C(dataset):C(compound)**	35.104799	48	3.370926	5.39E-12
**Residual**	114.770876	529	NaN	NaN

The variability due to compound structure is more important than the variability arising from different dataset, although the latter being significant. Given the large variability in logB observed for single compounds within *and* between datasets, we can still safely pool the measurements from different monitoring campaigns for the purpose of modelling structure-dependent WWTP breakthrough. For future studies, it would be interesting to focus more closely on the parameters that are responsible for the systematic differences between the monitoring campaigns and the WWTP, also considering that this batch effect might be affected by different molecular structures, as indicated by significant interaction effect of dataset x compound.

7 Descriptors

We calculated **PaDEL** descriptors using padelpy, **Mordred** descriptors using the Mordred calculator, **MACCS** fingerprints, Extended Connectivity fingerprints (**ECFP),** and **RDKit** Fingerprints using RDKit v2024.09.05 and enviPath biotransformation rules (**ePFP)** using enviPath-python v0.2.3 PaDEL and Mordred are molecular descriptors widely used in cheminformatics.[7,8] MACCS are substructure-based fingerprints that were chosen because they are easy to interpret. ECFPs are a type of circular fingerprints, meaning that substructures are generated iteratively around each atom, capturing a wider range of molecular features.[9] This makes Morgan fingerprints better suited for tasks that require more detailed molecular representations, such as similarity searching and structure-activity modeling.[10,11]

Additionally, we used one-hot encoding to represent functional groups involved in specific biological transformations. These are functional groups that triggered a biotransformation rule according to enviPath. enviPath is a prediction system for microbial transformations.[12] In this way, we created a substructure-based fingerprint similar to MACCS but with emphasis on substructures that relate to microbial transformation.

8 Nested cross validation

We used a nested cross validation as follows: in each fold the data is split in two subsets, one subset used for training and validation and one subset reserved for testing. These folds represent the “outer loop”. Inside each fold, the training-validation set is also split 5 times, this is the “inner loop” which is used for optimization of hyperparameters and for feature selection when applicable.

Hyperparameters were optimized for selected regressors, using nested 5-fold CV where the «inner loop» was scikit-learn's GridSearchCV. The list of hyperparameters selected for optimization for each regressor can be found in PEPPER's documentation. All features were scaled using MinMaxScaler (scikit-learn), quasi-constant features were removed with VarianceThreshold (scikit-learn), and highly correlated features were eliminated using an in-house method based on pairwise correlation coefficients using a cluster threshold of 0.01.

Furthermore, to account for the possibility that models fit to random noise we performed y-scrambling tests as described by Rücker et al. (2007)[13]. In their work, attention is called to carry out the full data analysis including descriptor selection. We gave full freedom to the model by repeating all feature selection and optimization steps and using the same training and testing sets as in the original model development workflow with the sole exception that logB values were scrambled to purposely "break" the structure-activity relationship. No correlation was observed, and every run led to extremely poor performance on unseen data $r2 < 0$ and $RMSE > 0.8$.

There is a subset of 462 molecules that fulfill all the curation criteria and thus it is the dataset with the highest confidence. We consider this subset as ideal for testing, but it represents a very large portion of the full set. So, for testing we divided this subset in 5 and we train models using 80% of this subset in addition to molecules that do not meet these criteria and we test the performance on the remaining 20%. Enabling us to evaluate on unseen data of the highest quality while maximizing the number of examples for training.

9 Preliminary models

We tested all combinations of regressors and descriptors using default hyperparameters and with 5-fold cross validation over the whole dataset with the purpose of understanding which combinations tended to perform better for our target endpoint. Figure S4. Performance of the different regressors tested. Multiple Linear Regression (MLR) using Ordinary Least Squares (OLS) was also tested but not included in the figure. The RMSE was above 10 and R^2 below -30. shows the performance of the different regressors tested. Multiple Linear Regression (MLR) using Ordinary Least Squares (OLS) was also tested but not included in the figure. The RMSE was above 10 and R2 below -30.

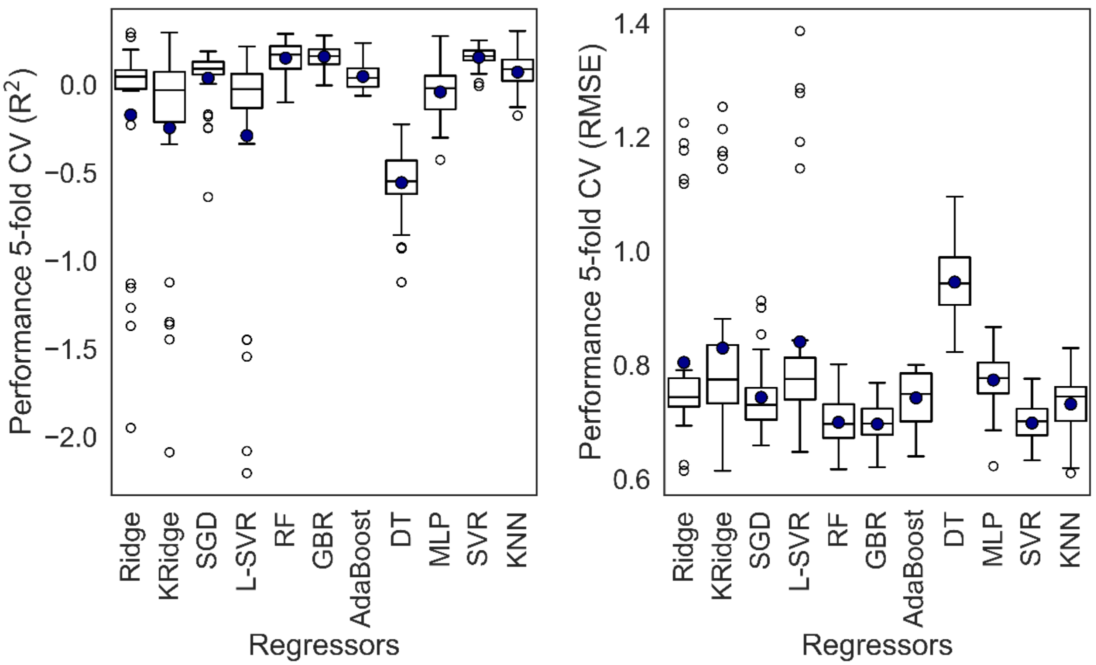

Figure S4. Performance of the different regressors tested. Multiple Linear Regression (MLR) using Ordinary Least Squares (OLS) was also tested but not included in the figure. The RMSE was above 10 and R^2 below -30.

10 Statistical Significance of the Differences in Performance for Different Regressors

When analyzing differences in performance, we first assessed normality using the Shapiro-Wilk test, which revealed evidence of departure from normality. Therefore, we applied the Friedman test, a non-parametric test suited for such cases, which indicated statistically significant differences among the regressors ($p < 10^{-10}$). To identify which pairs were significantly different, we conducted a repeated measures ANOVA with Bonferroni correction to account for non-normality. The results confirmed that GBR, RF, and SVR perform significantly better than KNN and AB. However, there was no evidence to single out any one of GBR, RF, or SVR as the optimal regressor.

To further investigate the impact of different features, we analyzed results for SVR, RF, and GBR together, as these regressors have shown the best performance. The Friedman test revealed statistically significant differences among features ($p < 10^{-5}$). We observed that all features, except for the enviPath-triggered-rules fingerprint (ePFP), have similar mean, median, and distribution. To confirm whether this apparent difference was statistically significant, we compared ePFP against other features using the Wilcoxon signed-rank test, which confirmed a significant difference ($p < 10^{-4}$).

11 Statistical Significance of the Differences in Performance when Training with Different subsets

After analyzing the performance of models trained with different subsets, there seems to be considerable variance, and the mean values for all strategies seemed similar, making it unclear whether any curation strategy was significantly better (Figure S6. Performance using different

data subsets for training when using additional curation criteria. The criteria are identified as follows: (I) Only data available in 3 WWTPs or more, (II) No substances with breakthrough above 120%, (III) No substances with large variability across WWTPs and (IV) only measurements above the limit of quantification (V) Remove volatile and highly sorbing substances.). The main motivation to explore criterium (I) is to asses whether adding new substances to the training set is useful even if there is a chance that these values are highly biased or incorrect which is more likely when only one or two observations are available as compared to 3 or more observations. The motivation to explore criterium (II) is that very high values can still be informative even if they are inaccurate. Very large breakthrough values may arise from uncertainty in the measurements but could also reflect actual formation during treatment, for example the increased concentration of metabolites during biological treatment. Disentanglement of actual formation from analytical uncertainty is extremely challenging and beyond the scope of this study; however, we note that formation of metabolites adds major complexities to QSAR modeling in this context. The motivation to explore criterium (III) is to systematically address the influence of variability in and within WWTPs. Figure S*5* shows the distribution of standard deviations for all chemicals, illustrating that for some substances the variability can be larger than 1 log unit. The vertical line shows the arbitrary threshold (i.e., 0.7) that we set to compare the effect of these large variabilities. We believe that this offers a good compromise by considerably limiting the overall variability without excluding too many substances. The motivations to explore criteria (IV) and (V) is to determine if allowing values below the LOQ and allowing highly sorbing and volatile substances could be beneficial to the model in efforts to cover a wider range of molecules in training set.

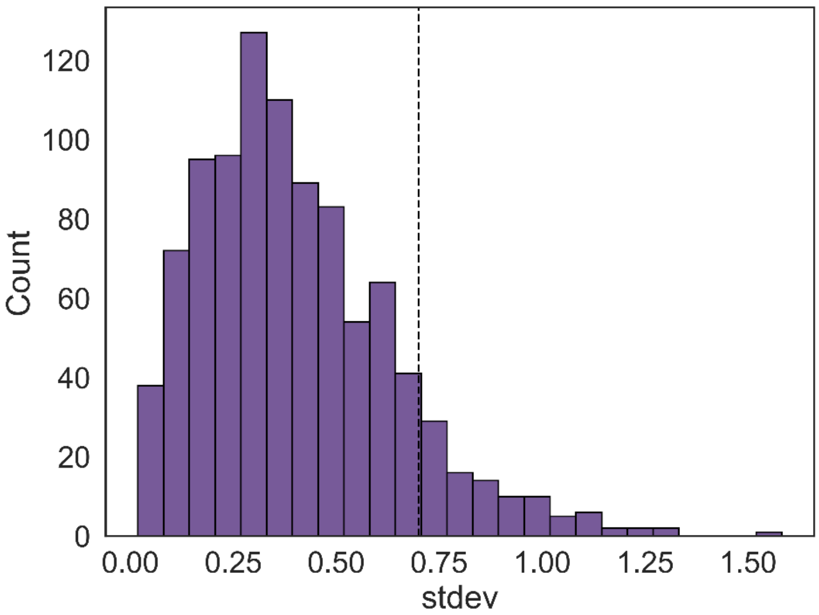

Figure S5. Distribution of the standard deviation of breakthrough values (in log scale) of individual molecules across the whole dataset. Each count represents one molecule and the standard deviation is calculated across the whole dataset.

To assess statistical significance, we conducted a Friedman test, first confirming a departure from normality with the Shapiro-Wilk test. The resulting p-value (0.805) of the Friedman test indicated no significant differences among curation strategies. We also tested for significant differences among curation strategies for each descriptor-regressor pair individually by conducting a repeated measures ANOVA after confirming normality and sphericity; however, the ANOVA revealed no significant differences. Judging by the mean R^2 there is a benefit of applying all criteria compared to no additional curation, even though the training size is three times smaller. There is an initial tendency of improved performance until combination (I+III). Most other sets have similar or worse performance except for the models trained with data which considers all curation criteria and the combination (I+II+IV+V). However, we opted for

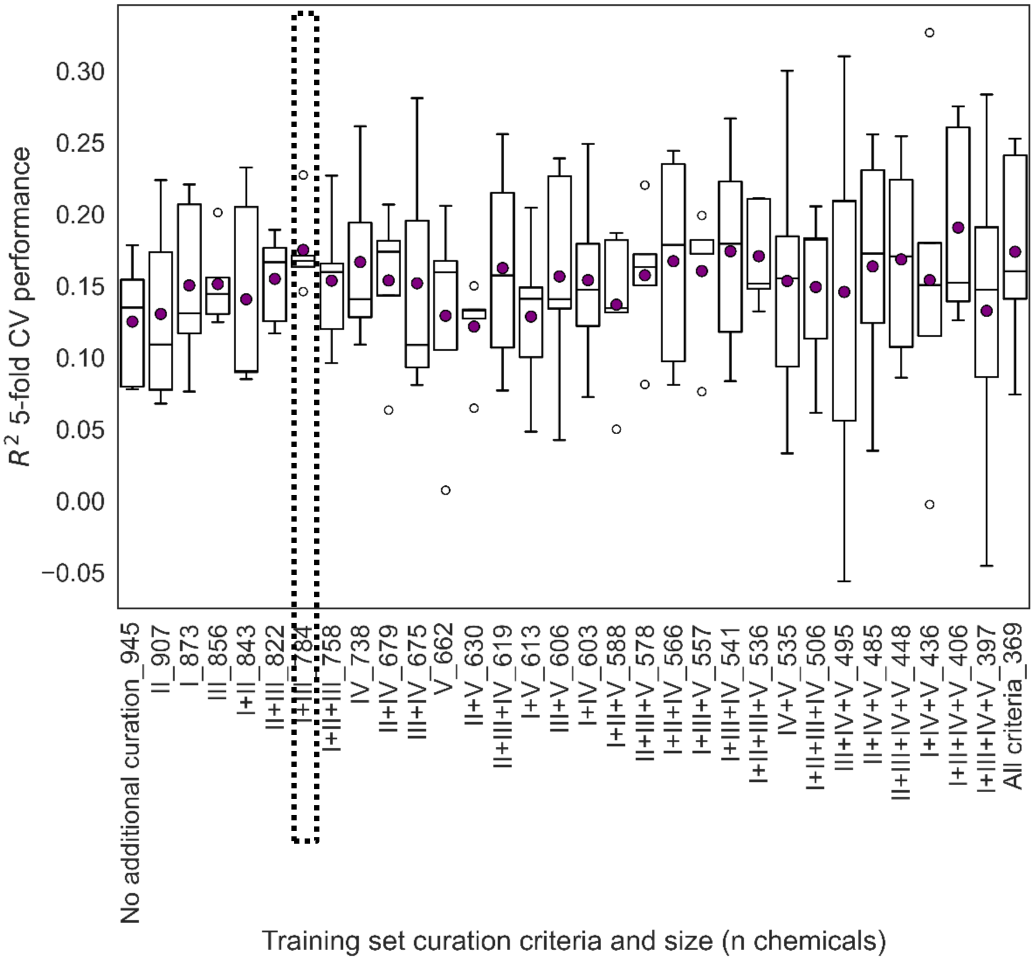

Figure S6. Performance using different data subsets for training when using additional curation criteria. The criteria are identified as follows: (I) Only data available in 3 WWTPs or more, (II) No substances with breakthrough above 120%, (III) No substances with large variability across WWTPs and (IV) only measurements above the limit of quantification (V) Remove volatile and highly sorbing substances. The number that follows the curation strategies is the number of chemicals that fulfill such criteria.

combination (I+III) for further model development considering that a larger number of chemicals (856 compared to 369) in the training set leads to a more general model, and we believe that restricting the domain of applicability does not compensate the small gain in performance. Finally, we also acknowledge that large variability across folds may obscure potential differences. As new data becomes available or as models improve, we expect more consistent

predictions across folds, potentially making the effects of different curation strategies more evident. Therefore, we recommend that this analysis be repeated periodically, and to support this, we have provided thorough documentation to facilitate reproducibility to reproduce this workflow in the PEPPER repository (github.com/FennerLabs/pepper).

12 Hyperparameter optimization & Bias correction

We optimized the hyperparameters of the random forest (RF) model in two steps. First, we conducted a random search across a large hyperparameter space, followed by a finer tuning using a grid search centered on the values identified in the random search. Both optimization strategies were performed using 5-fold cross-validation.

In selecting hyperparameters, we aimed to explore simpler models by including a limited range

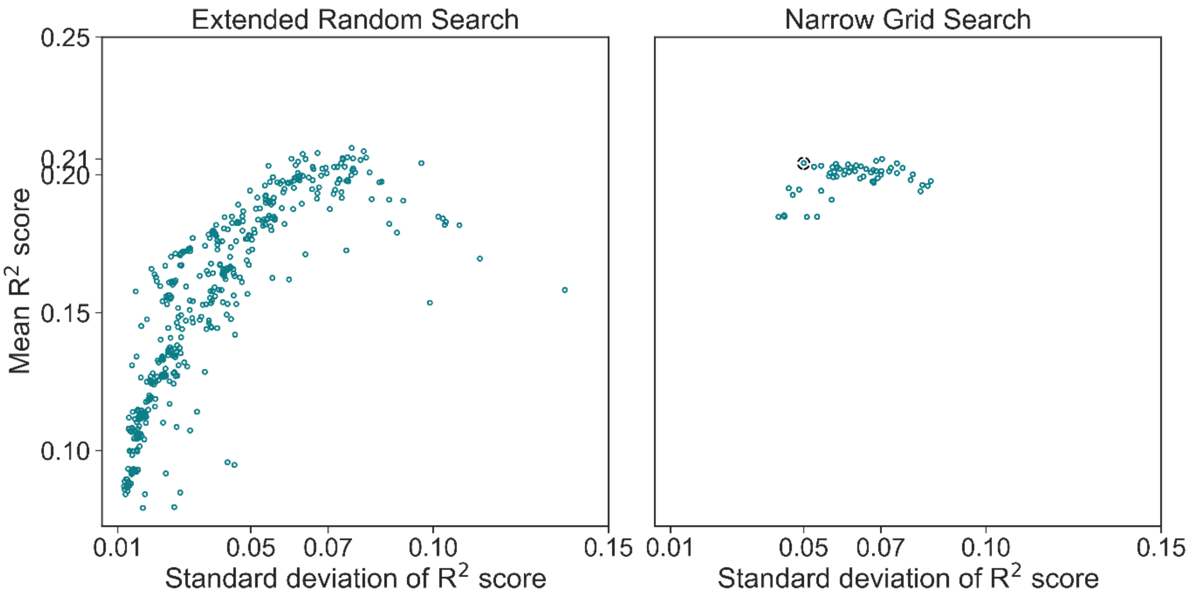

Figure S7. Initial randomized search and b) narrower grid search to select the best hyperparameters using 5-fold cross validation. The y-axis represents the mean scores of the cross validation for a given combination of hyperparameters and the x-axis represents the standard deviation of the scores among folds. We consider that a lower standard deviation is evidence of a more robust model, so we selected the hyperparameters that yielded the best score with the lowest standard deviation.

of tree numbers (from 10 to 300 in increments of 10) and shallow maximum depths (starting from 5, in increments of 5). For further tuning, we considered larger values for min_samples_split and min_samples_leaf (up to 20, in steps of 2) and smaller values for max_features, specifically the square root and log2 of n_samples. Bootstrapping was used in all cases to reduce the risk of overfitting. To select the best-performing models, we prioritized those with the highest R^2 scores and the lowest standard deviation across folds, aiming for robust models during cross-validation. Figure S7. Initial randomized search and b) narrower grid search to select the best hyperparameters using 5-fold cross validation. The y-axis represents the mean scores of the cross validation for a given combination of hyperparameters and the x-axis represents the standard deviation of the scores among folds. We consider that a lower standard deviation is evidence of a more robust model, so we selected the hyperparameters that yielded the best score with the lowest standard deviation..a shows the R^2 scores and standard deviations for 500 candidate models from the random search and Figure S7. Initial randomized search and b) narrower grid search to select the best hyperparameters using 5-fold cross validation. The y-axis represents the mean scores of the cross validation for a given combination of hyperparameters and the x-axis represents the standard deviation of the scores among folds. We consider that a lower standard deviation is evidence of a more robust model, so we selected the hyperparameters that yielded the best score with the lowest standard deviation..b shows 1,080 candidate models from the grid search; note that each circle represents a candidate model but many had identical performance. In the grid search, hyperparameters were adjusted in finer increments around the values identified in the random search (e.g., if n_trees from the random search was 60, then grid search values would include [50, 55, 60, 65, 70]). The search was expanded if boundary values were optimal and continued until no further improvement was

observed. The set of optimal hyperparaters for the best model are summarized in Table S*4* , and can also be found in the pepper-lab repository as "regressor_settings_singlevalue_wwtp_optimized.yml"

Table S4. Set of optimal parameters for the best model.

Parameter name	Optimized value
n_estimators	40
max_depth	40
min_samples_split	14
min_samples_leaf	7
max_features	1.0 (=all features)
random_state	42
n_jobs	-1
Note: for other parameters values are default values for scikit-learn 1.6.1	

13 Applicability domain & Confidence metrics

Figure S8. Model performance as a function of confidence in predictions using similarity metrics and ensemble prediction agreement metrics. Using the standard deviation in the predictions of the individual trees (TreeSD) is a good measure of confidence as it leads to lower

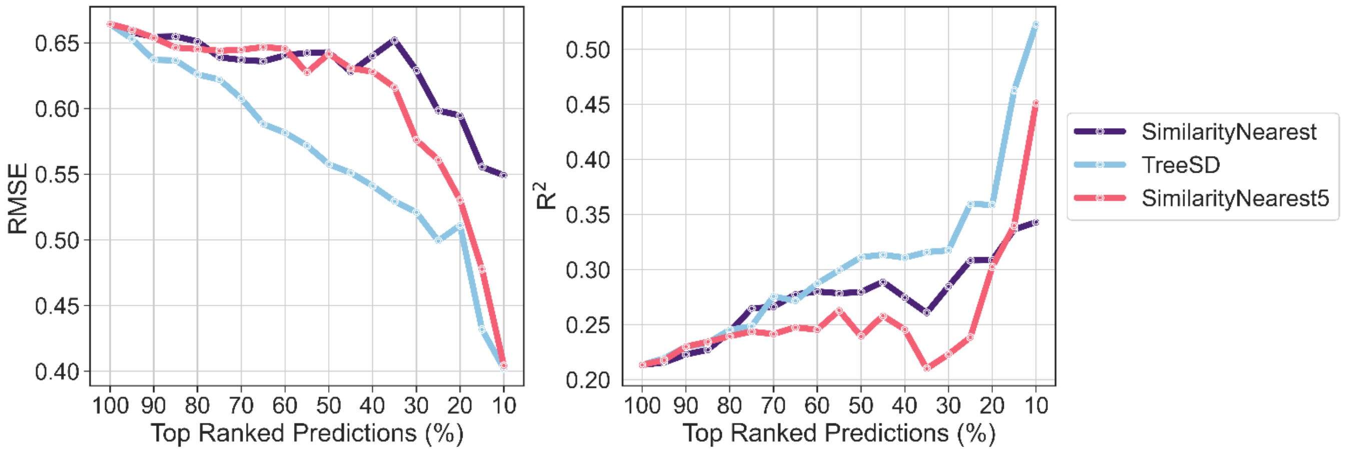

Figure S8. Model performance as a function of confidence in predictions using similarity metrics and ensemble prediction agreement metrics. Using the standard deviation in the predictions of the individual trees (TreeSD) is a good measure of confidence as it leads to lower errors.

errors. shows changes in performance as a function of different levels of confidence in prediction according to 3 different criteria, SimilartyNearest, SimilarityNearest5 and TreeSD. The figures demonstrate that a RMSE close to 0.4 and R^2 close to 0.5 can be achieved for those predictions in which the individual trees closely agree, that is, TreeSD is very small. The raw values are shown in Figure S*9* which suggest TreeSD less than 0.3 is ideal.

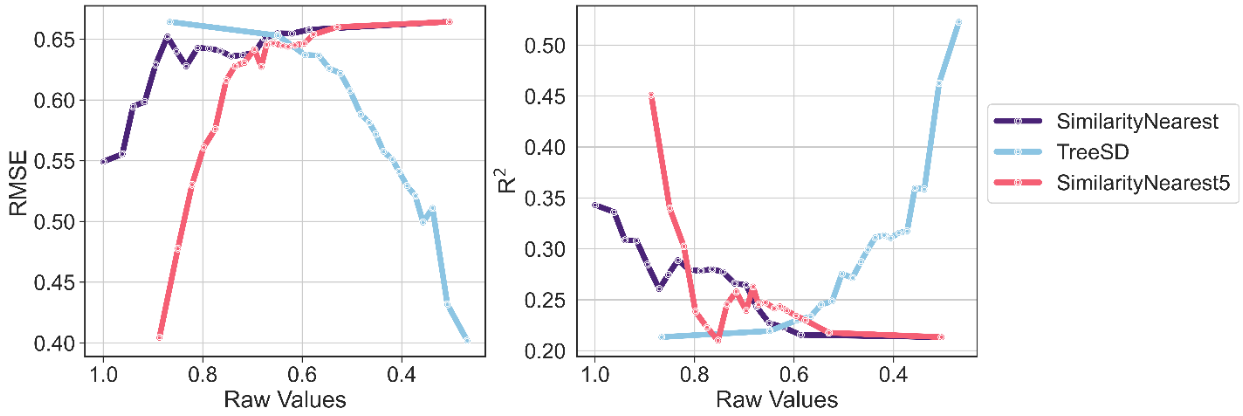

Figure S9. Raw values that correspond with the ranked percentage values in the previous figure. This allows visualization of the actual thresholds and their expected performance. Note that the x-axis is reversed. The interpretation is that in general smaller TreeSD leads to lower error and higher R2 and the opposite for Similarity metrics where decreased similarity ends to result in worse performance (higher RMSE and lower R2).

For the pepper-app (pepper-app.streamplit.app). we established a confidence score based on TreeSD observed during training: a confidence score of 0 is assigned when the prediction's standard deviation exceeds the maximum observed during training, and a score of 1 when the standard deviation is below the minimum observed. Intermediate values are linearly scaled

within this range. Thus, if one required to set a clear boundary for the domain of applicability, one can interpret a confidence score of 0 as an indication that a molecule falls outside the model's domain of applicability. Moreover, based on our cross-validation results, users can expect errors around 0.5 log units and a correlation coefficient exceeding 0.4 for predictions with a confidence score close to 1.

Furthermore, to better contextualize the performance metrics obtained we simulated the "best achievable performance" by randomly choosing values from the experimental data to simulate predictions with an accuracy equivalent to experimental data. The results of the simulation are shown in Figure S*10*.

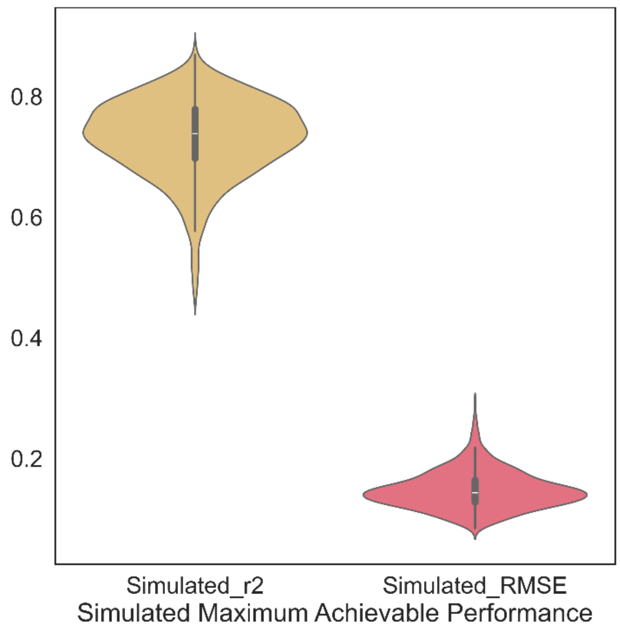

Figure S10. Simulated maximum achievable performance by simulating a model that predicts values from the actual experimental values. Conceptually it illustrates a model with an accuracy that matches experimental variability.

14 Analysis of predictions using STPWIN

STPWIN provides an estimate of breakthrough by combining several mechanisms including volatilization and biodegradation. Figure S11.a shows the agreement between measured and predicted breakthrough and as a color code the fraction of total removal that is attributed to biodegradation. That is, blue circles are substances that were removed almost entirely by biodegradation while red circles represent substances for which biodegradation played only a minimal role (i.e., <10% of the total removal). As expected, nearly all predictions rely heavily on biodegradation, indicating that prediction accuracy depends largely on the accuracy of the primary biodegradation rate constant. Similarly, Lautz et al.[14] observed that errors were 10 times higher when using biodegradation rate constants predicted by BIOWIN in comparison to using measured rate constants. Their study also confirmed that using plant-specific reactor parameters did not improve predictions significantly compared to simply using default values, which also highlight the enormous weight of biodegradation rate constants in the prediction errors.

We also observed that many STPWIN predictions fall into three distinct bins: high (close to 0 log units), intermediate (around -0.5 log units), and low (approximately -1.0 log units) breakthrough values. This "binning" effect results from the nature of BIOWIN predictions, which output semi-quantitative but effectively still categorial values. From Figure S11, it can further be appreciated that these same values were incorrectly predicted for many compounds effectively exhibiting a large range of different observed breakthrough values. Additionally, for molecules where biodegradation plays a smaller role in total removal according to STPWIN (biodegradation/total removal ratio < 0.4), STPWIN predictions were more accurate Figure S11.b.

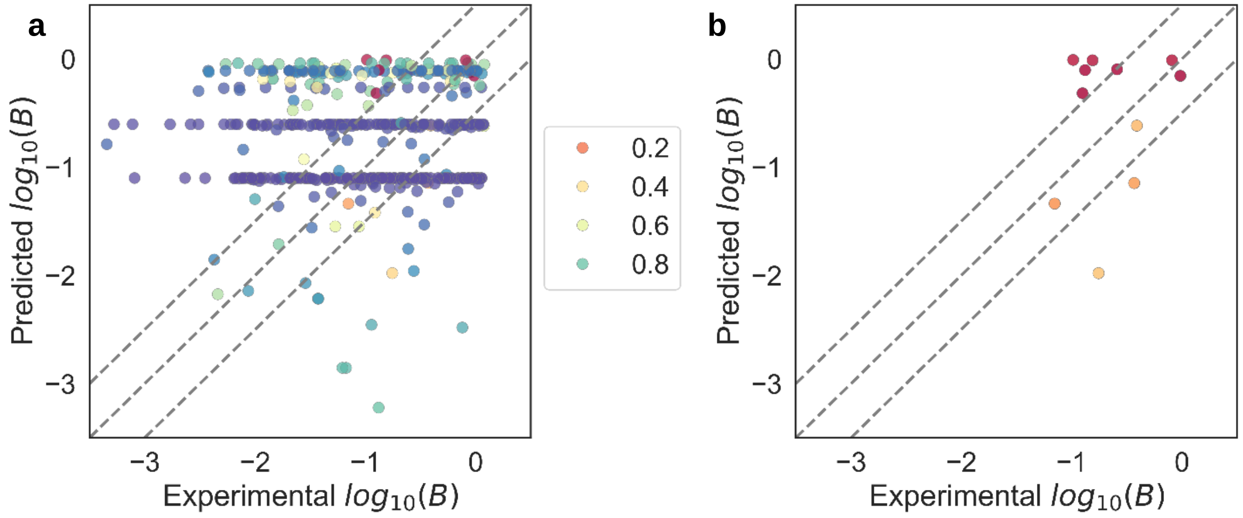

Figure S11. Predictions STPWIN. Predictions of STPWIN. a) The color code represents the fraction of total removal attributed to biodegradation. b) Predictions for chemicals where biodegradation represents less than 40% of the total removal

15 References

(1) Munkhammar, V. *Evaluation of Pharmaceutical Removal in Seven WWTPs : Efficiency of Different Treatment Technologies*; 2023.

(2) S. McLachlan, M.; Li, Z.; Jonsson, L.; Kaserzon, S.; W. O'Brien, J.; F. Mueller, J. Removal of 293 Organic Compounds in 15 WWTPs Studied with Non-Targeted Suspect Screening. *Environmental Science: Water Research & Technology* **2022**, *8* (7), 1423–1433. https://doi.org/10.1039/D2EW00088A.

(3) Kessler, C. Master Thesis_SNF_project, University of Zèrich, 2023.

(4) Kalt, M. Breaking It Down: Unveiling the Roles of Chemical Structures, Micropollutant Load and Treatment Technology on Biotransformation in Activated Sludge. *Environ. Sci. Technol.* **2025**, *(in preparation)*.

(5) Ternes, T. A.; Joss, A.; Siegrist, H. Peer Reviewed: Scrutinizing Pharmaceuticals and Personal Care Products in Wastewater Treatment. *Environ. Sci. Technol.* **2004**, *38* (20), 392A-399A. https://doi.org/10.1021/es040639t.

(6) Achermann, S.; Falås, P.; Joss, A.; Mansfeldt, C. B.; Men, Y.; Vogler, B.; Fenner, K. Trends in Micropollutant Biotransformation along a Solids Retention Time Gradient. *Environ. Sci. Technol.* **2018**, *52* (20), 11601–11611. https://doi.org/10.1021/acs.est.8b02763.

(7) Moriwaki, H.; Tian, Y.-S.; Kawashita, N.; Takagi, T. Mordred: A Molecular Descriptor Calculator. *Journal of Cheminformatics* **2018**, *10* (1), 4. https://doi.org/10.1186/s13321-018-0258-y.

(8) Yap, C. W. PaDEL-Descriptor: An Open Source Software to Calculate Molecular Descriptors and Fingerprints. *Journal of Computational Chemistry* **2011**, *32* (7), 1466–1474. https://doi.org/10.1002/jcc.21707.

(9) Capecchi, A.; Probst, D.; Reymond, J.-L. One Molecular Fingerprint to Rule Them All: Drugs, Biomolecules, and the Metabolome. *Journal of Cheminformatics* **2020**, *12* (1), 43. https://doi.org/10.1186/s13321-020-00445-4.

(10) Gao, K.; Duy Nguyen, D.; Sresht, V.; M. Mathiowetz, A.; Tu, M.; Wei, G.-W. Are 2D Fingerprints Still Valuable for Drug Discovery? *Physical Chemistry Chemical Physics* **2020**, *22* (16), 8373–8390. https://doi.org/10.1039/D0CP00305K.

(11) Lee, S.; Lee, M.; Gyak, K.-W.; Kim, S. D.; Kim, M.-J.; Min, K. Novel Solubility Prediction Models: Molecular Fingerprints and Physicochemical Features vs Graph Convolutional Neural Networks. *ACS Omega* **2022**, *7* (14), 12268–12277. https://doi.org/10.1021/acsomega.2c00697.

(12) Hafner, J.; Lorsbach, T.; Schmidt, S.; Brydon, L.; Dost, K.; Zhang, K.; Fenner, K.; Wicker, J. Advancements in Biotransformation Pathway Prediction: Enhancements, Datasets, and Novel Functionalities in enviPath. *Journal of Cheminformatics* **2024**, *16* (1), 93. https://doi.org/10.1186/s13321-024-00881-6.

(13) Rücker, C.; Rücker, G.; Meringer, M. Y-Randomization and Its Variants in QSPR/QSAR. *J. Chem. Inf. Model.* **2007**, *47* (6), 2345–2357. https://doi.org/10.1021/ci700157b.

(14) Lautz, L. S.; Struijs, J.; Nolte, T. M.; Breure, A. M.; van der Grinten, E.; van de Meent, D.; van Zelm, R. Evaluation of SimpleTreat 4.0: Simulations of Pharmaceutical Removal in Wastewater Treatment Plant Facilities. *Chemosphere* **2017**, *168*, 870–876. https://doi.org/10.1016/j.chemosphere.2016.10.123.
